# Data-Driven Analysis of COVID-19 Reveals Persistent Immune Abnormalities in Convalescent Severe Individuals

**DOI:** 10.3389/fimmu.2021.710217

**Published:** 2021-11-19

**Authors:** Jackwee Lim, Kia Joo Puan, Liang Wei Wang, Karen Wei Weng Teng, Chiew Yee Loh, Kim Peng Tan, Guillaume Carissimo, Yi-Hao Chan, Chek Meng Poh, Cheryl Yi-Pin Lee, Siew-Wai Fong, Nicholas Kim-Wah Yeo, Rhonda Sin-Ling Chee, Siti Naqiah Amrun, Zi Wei Chang, Matthew Zirui Tay, Anthony Torres-Ruesta, Norman Leo Fernandez, Wilson How, Anand Kumar Andiappan, Wendy Lee, Kaibo Duan, Seow-Yen Tan, Gabriel Yan, Shirin Kalimuddin, David Chien Lye, Yee-Sin Leo, Sean W. X. Ong, Barnaby E. Young, Laurent Renia, Lisa F. P. Ng, Bernett Lee, Olaf Rötzschke

**Affiliations:** ^1^ Singapore Immunology Network, Agency for Science, Technology and Research, Singapore, Singapore; ^2^ A*STAR Infectious Disease Labs, Agency for Science, Technology and Research, Singapore, Singapore; ^3^ Department of Biological Sciences, National University of Singapore, Singapore, Singapore; ^4^ Department of Biochemistry, Yong Loo Lin School of Medicine, National University of Singapore, Singapore, Singapore; ^5^ Department of Infectious Diseases, Changi General Hospital, Singapore, Singapore; ^6^ Department of Medicine, National University Hospital, Singapore, Singapore; ^7^ Department of Infectious Diseases, Singapore General Hospital, Singapore, Singapore; ^8^ Emerging Infectious Diseases Program, Duke-NUS Medical School, Singapore, Singapore; ^9^ National Centre for Infectious Diseases, Singapore, Singapore; ^10^ Department of Infectious Diseases, Tan Tock Seng Hospital, Singapore, Singapore; ^11^ Lee Kong Chian School of Medicine, Nanyang Technological University, Singapore, Singapore; ^12^ Yong Loo Lin School of Medicine, National University of Singapore and National University Health System, Singapore, Singapore; ^13^ Saw Swee Hock School of Public Health, National University of Singapore and National University Health System, Singapore, Singapore; ^14^ Institute of Infection, Veterinary and Ecological Sciences, University of Liverpool, Liverpool, United Kingdom

**Keywords:** COVID-19, cytokine profile, severity, SARS – CoV – 2, inflammation, active infection, immunophenotyping, immune recovery

## Abstract

Severe SARS-CoV-2 infection can trigger uncontrolled innate and adaptive immune responses, which are commonly associated with lymphopenia and increased neutrophil counts. However, whether the immune abnormalities observed in mild to severely infected patients persist into convalescence remains unclear. Herein, comparisons were drawn between the immune responses of COVID-19 infected and convalescent adults. Strikingly, survivors of severe COVID-19 had decreased proportions of NKT and Vδ2 T cells, and increased proportions of low-density neutrophils, IgA+/CD86+/CD123+ non-classical monocytes and hyperactivated HLADR+CD38+ CD8+ T cells, and elevated levels of pro-inflammatory cytokines such as hepatocyte growth factor and vascular endothelial growth factor A, long after virus clearance. Our study suggests potential immune correlates of “long COVID-19”, and defines key cells and cytokines that delineate true and quasi-convalescent states.

## Introduction

Severe COVID-19 is caused by severe acute respiratory syndrome coronavirus 2 (SARS-CoV-2) that affects about 5-15% of infected individuals (1, 2). Clinical presentation is highly variable, whereby most patients (about 80%) experience mild to moderate symptoms, and some individuals develop pneumonia, acute respiratory distress syndrome, septic shock or even multiple organ failure from a hyperactivated immune system (3, 4). Previous studies of peripheral blood mononuclear cells (PBMCs) from COVID-19 patients using cytometry and transcriptomic methods have revealed changes in several cellular immunotypes, including monocytes, natural killer cells, dendritic cells, neutrophils and T cells (5–9). In patients with severe COVID-19, immune perturbations are characterized by advanced lymphopenia in the T cell compartment, elevated immature neutrophils and altered myeloid cell frequencies (5–9). Another hallmark of severe COVID-19 is the cytokine storm associated with elevated levels of cytokines (IL1β, IL1Rα, IL2, IL6, IL7, IL10, G-CSF, TNFα), chemokines (IP10, MCP1, MIP1α) and endogenous neutrophil calprotectin (8, 10, 11). Although these studies have provided deeper insights on COVID-19 induced immunopathology, the long-term complications of COVID-19 remain unclear. This is important as a segment of COVID-19 survivors, including those who had mild disease continued to experience symptoms or become disease vulnerable (12).

Upon clearance of SARS-CoV-2, a sizeable number of recovered patients may exhibit lingering immune responses, which are believed to be responsible for long-COVID symptoms, including extreme fatigue, brain fogs and depression (13, 14). Ongoing inflammatory processes or aberrant immune responses can collectively contribute to clinical manifestations due to cellular and molecular damages of COVID-19 (15). At the same time, it is important to fully characterize the contribution of many immune abnormalities from early COVID-19 infection leading into convalescence, and the return to baseline. Yet, a comprehensive insight into the immunopathology of ongoing immune dysfunctions is still missing.

Previously, we have shown the neutrophil to Vδ2 ratio as prognostics for COVID-19 severity (7), as well as massive cytokine storm (11, 16). Also, we have reported protective mechanisms among asymptomatic patients (17). In this study, given the complex clinical manifestations, detailed analyses of the course of COVID-19 until convalescence were performed using high-parameter mass cytometry and multiplexed plasma cytokine profiling. We show that COVID-19 continues to alter the immune system following virus clearance; (i) COVID-19 severity is characterized by abundant low-density neutrophils or high neutrophil-to-lymphocyte ratio persisting into convalescence, (ii) elevated levels of hyperactivated CD8^+^ T cells and proinflammatory non-classical monocytes were found in the blood among convalescent severe COVID-19 patients, and (iii) high values of HGF, VEGF-A and TNF-α are associated with COVID-19 severity leading into convalescence. Overall, we have performed a detailed evaluation of more than 5000 immunotypes from both adaptive and innate branches along with a range of circulating cytokines and chemokines, to chart the most significant changes in the immune system that persisted into convalescence, which suggest incomplete recovery, and identified pathways for therapeutic agents.

## Materials and Methods

### Study Design, Sample Size and Participants

For this study, 77 COVID-19 patients and 10 healthy donors were recruited between April and June 2020. Enrollment of COVID-19 patients was *via* PROTECT, a Singapore COVID-19 cohort study among seven public health institutions. Healthy individuals were recruited under a Singapore Immunology Network study entitled, “Study of blood cell subsets and their products in models of infection, inflammation and immune regulation”. Both studies had received prior approval from their respective institutional review boards (IRBs). All individuals involved in this study were over the age of 21, comprising 66 males and 21 females. Additional demographic details can be found in **Supplementary Table 1**.

### Sample Collection

Blood from healthy adult donors and COVID-19 patients were collected in BD Vacutainer CPT Tubes and processed according to manufacturer’s instructions to obtain the PBMC and plasma fractions. Isolated PBMCs were then used for mass cytometry staining after two washes with 1X phosphate buffer saline (PBS).

### Cytometry by Time-of-Flight (CyTOF) Sample Processing and Data Acquisition

Freshly isolated ficoll-density centrifuged PBMCs were plated at 0.5 – 1 x 10^6^ in a 96-well V bottom plates and stained for viability with 100 µL of 66 µM of cisplatin (Sigma-Aldrich) for 5 minutes on ice. Cells were then washed with staining buffer (4% v/v fetal bovine serum, 0.05% v/v sodium azide in 1X PBS) and stained with anti-γδTCR-PE and anti-Vδ1-FITC in 50 µL reaction volume for 15 minutes at room temperature. Cells were washed with staining buffer and then stained with 50 µL of metal isotope-labeled surface antibodies on ice. After 20 minutes, cells were washed with staining buffer, followed by PBS, and fixed in 4% v/v paraformaldehyde (PFA, Electron Microscopy Sciences) at 4°C overnight. On the following day, cells were incubated in staining buffer for 5 minutes. Cellular DNA was labeled at room temperature with 170 nM iridium intercalator (Fluidigm) in 2% v/v PFA/PBS. After 20 minutes, cells were washed twice with staining buffer.

Prior to CyTOF acquisition, cells were washed twice with water before final re-suspension in water. Cells were enumerated, filtered and diluted to a final concentration of 0.6 x 10^6^ cells/mL. EQ Four Element Calibration Beads (Fluidigm) were added to the samples at a final concentration of 2% v/v prior to acquisition. Samples were acquired on a Helios Mass Cytometer (Fluidigm) at an event rate of < 500 events per second. After CyTOF acquisition, data were exported in flow-cytometry (FCS) format, normalized to 300,000 PBMCs and events with parameters having zero values were randomized using a uniform distribution of values between minus-one and zero. Subsequently, manual gating was performed to exclude residual beads, debris and dead cells.

### Gating Strategy for CyTOF

We have designed a 40-plex antibodies panel for mass cytometry and performed non-supervised Uniform Manifold Approximation and Projection (UMAP) or Triplet-constraint (TriMAP) dimensionality reduction for larger dataset embedding of ficoll-density centrifuged PBMCs obtained from both COVID-19 active and convalescent patients (18, 19). Iterative manual and UMAP clustering identified populations of T cells, B cells, monocytes (Mono), natural killer (NK) cells, dendritic cells (DCs), innate lymphoid cells (ILCs), mucosal-associated invariant T (MAIT) cells, basophil as well as the low-density (LD) neutrophils based on their cell surface expression markers to generate 327 different immune cell subpopulations.

### Multiplex Microbead-Based Luminex Immunoassays

Plasma samples were treated by solvent/detergent based on Triton™ X-100 (1%) for virus inactivation (20). Immune mediator levels in COVID-19 patient plasma across different active and convalescent groups were measured with 24-plex Human ProcartaPlex™ (ThermoFisher Scientific). The kit analyte detection panel included brain-derived neurotrophic factor (BDNF), beta-nerve growth factor (bNGF), hepatocyte growth factor (HGF), monocyte chemoattractant protein (MCP) 1, macrophage inflammatory protein (MIP) 1α, MIP1β, RANTES (regulated on activation, normal T cell expressed and secreted), stromal cell-derived factor 1 (SDF1α), interferon (IFN) gamma-induced protein 10 (IP10), IFNγ, interleukin (IL) IL1β, IL1RA, IL2, IL5, IL6, IL7, IL18, IL12p70, leukemia inhibitory factor (LIF), stem cell factor (SCF), tumor necrosis factor (TNFα), vascular endothelial growth factor A (VEGF-A), platelet derived growth factor (PDGF-BB), and placental growth factor (PLGF1).

Plasma from COVID-19 patients, healthy controls, as well as standards were incubated with fluorescent-coded magnetic beads pre-coated with respective antibodies in a black 96-well clear-bottom plate overnight at 4°C. After incubation, plates were washed 5 times with wash buffer (PBS with 1% v/v bovine serum albumin (Capricorn Scientific) and 0.05% v/v Tween-20 (Promega)). Sample-antibody-bead complexes were incubated with biotinylated detection antibodies for 1 hour and washed 5 times with wash buffer. Subsequently, Streptavidin-PE was added and incubated for another 30 minutes. Plates were washed 5 times again, before sample-antibody-bead complexes were re-suspended in sheath fluid for acquisition on the FLEXMAP^®^ 3D (Luminex) using xPONENT^®^ 4.0 (Luminex) software. Data analysis was done on Bio-Plex Manager™ 6.1.1 (Bio-Rad). Standard curves were generated with a 5-PL (5-parameter logistic) algorithm, reporting values for both mean florescence intensity (MFI) and concentration data.

Internal control samples were included in each plate to remove any potential plate effects. Readouts of these samples were then used to normalize the assayed plates. A correction factor was obtained from the median concentration values observed across the multiple assay plates and this correction factor was then used to normalize all the samples. The concentrations were logarithmically transformed to ensure normality. Analytes that were not detectable in patient samples were assigned the value of logarithmic transformation of the Limit of Quantification (LOQ).

### Multiplex Microbead-Based Quanterix Immunoassays

Plasma immune mediator levels in selected active and convalescence phase of COVID-19 patients were measured using SIMOA Cytokine 3-Plex B (C3PB) assay kit (Quanterix) and SIMOA IFN-a assay kit (Quanterix). C3PB kit analyte detection included interleukin (IL) IL6, IL17A and tumor necrosis factor α (TNFα).

Standards and plasma from COVID-19 patients and healthy controls were pre-diluted in a 96-well plate before loading into the Simoa^®^ HD-1 Analyzer (Quanterix) for data acquisition. Reagents from the C3PB and IFNα assay kits were prepared according to the kit manual and loaded into the analyzer. Fully automated data acquisition was done on Simoa^®^ HD-1 Analyzer (Quanterix). Standard curves were generated with a 4-PL (4-parameter logistic) algorithm, reporting values for concentration data.

### Quantification and Statistical Analysis

Active and convalescence phase samples were defined by PCR positivity and serve as time based clinical end points. Active COVID-19 infection can last up to a month, and samples were further divided into early (post illness onset, PIO <= 14 days) and late (PIO > 14 days). Convalescence phase samples were also further divided into early (PIO <= 28 days) and late (PIO > 28 days). At each time point, clinical data, whole blood and plasma were collected for all patients. The patients were screened for co-infections, and one patient was excluded due to HIV-1 positivity. Whole blood was immediately processed to isolate PBMCs using ficoll-density gradient centrifugation for mass cytometry, and plasma kept at -80°C for long-term storage.

**Figure 1:**
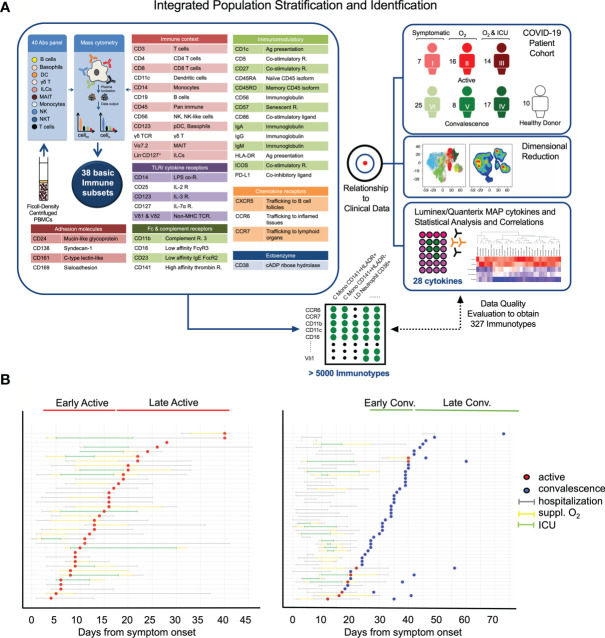
Study design and clinical characteristics of the cohort. **(A)** Schematic showing the pipeline for sample acquisition and analysis. A list of the antibody targets is presented. **(B)** Timelines for individual COVID-19 cases, indicating points of sample collection and any clinically pertinent detail e.g. duration of hospitalization, oxygen supplementation and admission to the intensive care unit (ICU). Patients are grouped as a function of days post illness onset (PIO) – d9 (early active; median: 9 days PIO), d20 (late active; median: 20 days PIO), d25 (early convalescence; median: 25 days PIO), d39 (late convalescence; median: 39 days PIO).

Severity based clinical end points were defined for active and convalescence phase samples separately. Three severity groups were defined for each phase consisting of symptomatic patients, patients requiring oxygen supplementation and patients requiring oxygen supplementation and awarded into intensive care unit as shown in **Figure 1A**.

Mass cytometry and cytokine measurements were associated to the clinical end points (time based as well as severity based) using Kruskal-Wallis tests followed by Dunn’s *post hoc* tests. Correlations between mass cytometry and cytokine measurements were done using Spearman Rank correlations. In the event that multiple samples from the same patient were available for same time period, the earliest of the samples was used for analyses to ensure that all samples used in the analyses are distinct. Multiple testing correction was done using the method of Benjamini and Hochberg. P values less than 0.05 were deemed to be significant. All statistical tests were two-sided (when appropriate) unless otherwise indicated. Statistical analyses were done using the R statistical language version 3.6.2. All statistical details are provided in the interactive viewers provided at https://data.mendeley.com/datasets/467s57xj8s/draft?a=15341765-e712-4eec-8107-a1d9c8da331a.

Overviews of the mass cytometry immune cell subpopulations were generated using UMAP in R version 3.6.2 using the ‘uwot’ package. Heatmaps were generated in R version 3.6.2 using the CompexHeatmap package. Graphs of the significant associations were generated in R version 3.6.2 using the iGraph package and visualized in Cytoscape version 3.8.0. Additional visualizations were done in TIBCO Spotfire.

### Data and Code Availability

Data generated and/or analyzed during this study are available in the following public repositories and also at https://data.mendeley.com/datasets/467s57xj8s/draft?a=15341765-e712-4eec-8107-a1d9c8da331a.

**Figure 2:**
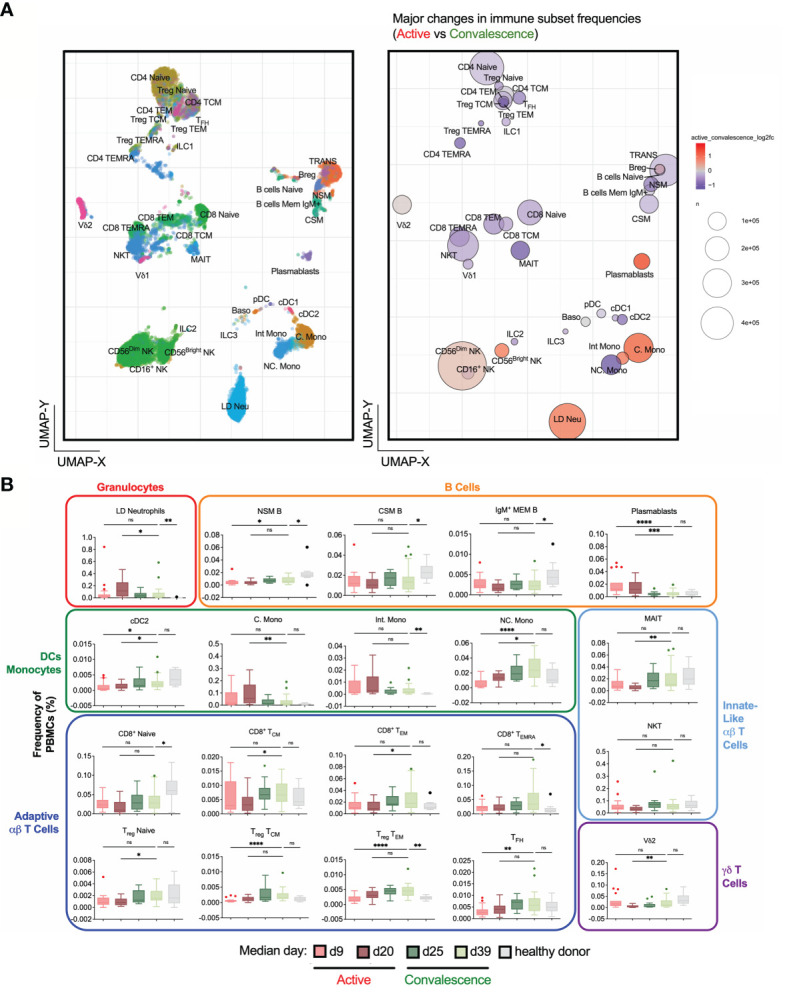
Frequency changes in 38 basic immune cell populations with SARS-CoV-2 infection. **(A)** Uniform Manifold Approximation and Projection (UMAP) plots of 38 main immune cell populations detected by mass cytometry (left). Right: Bubble representation of fold changes in the detected populations during activeinfection relative to convalescence, with color-coding done on a log2 scale, and bubble size reflecting the percentage of the subset. **(B)** Frequency-time plots of immune cell populations of interest over the course of disease. Asterisks indicate statistical significance- ns, not significant, *p < 0.03; **p < 0.002; ***p < 0.0002, ****p < 0.0001 (Kruskal-Wallis test with multiple comparison corrected on each disease phases versus healthy controls).

**Figure 3:**
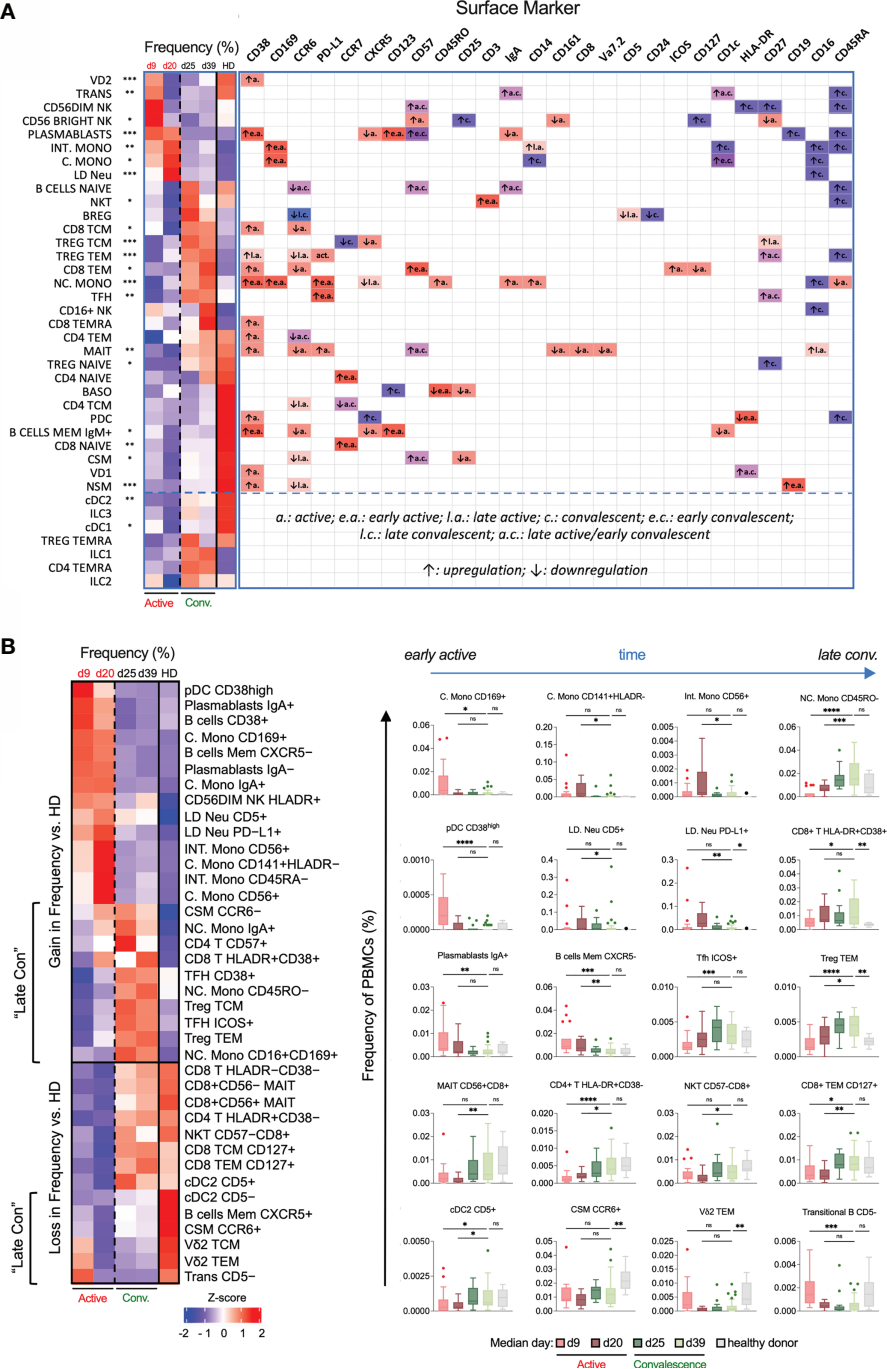
Temporal changes in frequencies and surface marker expression profiles of various immunotypes during active and convalescent COVID-19. **(A)** Left: Heatmap of CyTOF data of the frequencies of all 38 basic immune cell populations as a function of days post illness onset (PIO) – d9 (early active), d20 (late active), d25 (early convalescence) and d39 (late convalescence). Asterisks indicate statistical significance - *p < 0.05; **p < 0.01; ***p < 0.001 (Kruskal-Wallis test with multiple comparison corrected on all disease phases and healthy controls). Right: up- or down-regulation of indicated surface markers for the 38 main immune cell populations as a function of disease phase. **(B)** Left: Heatmap of CyTOF data of the frequencies of the top 38 immunotypes as a function of disease phase. Right: Box-and-whiskers plots of select immunotypes showing the frequency-time relationships, with mean and IQR indicated. “Late Con” refers to a group of immunotypes, which fail to recover to healthy levels even in late convalescence as post-infection aberrations. Asterisks indicate statistical significance- ns, not significant, *p < 0.03; **p < 0.002; ***p < 0.0002, ****p < 0.0001 (Kruskal-Wallis test with multiple comparison corrected on each disease phases versus healthy controls).

An interactive viewer of the mass cytometry data associations with clinical endpoints (**Figures 2A**, **3**, **5A** and **Supplementary Figure 2**) are available at https://www.dropbox.com/s/wz93vwn2vvjsjry/cytof_sample_group_association_results_paper_vis_covid19_cytof_results_viewer.html?dl=1.

An interactive viewer of the cytokine data associations with clinical endpoints (**Figures 6A, B**) are available at https://www.dropbox.com/s/4v107l3b65h5qfh/luminex_sample_group_association_results_vis_covid19_cytof_results_viewer.html?dl=1.

The mass cytometry, cytokine and clinical data is available as an Excel file at https://www.dropbox.com/s/yd2spn3lholhuv3/all_cytof_multimodal_data_paper.xlsx?dl=1.

An interactive viewer of interaction network in **Figure 7A** is available at https://www.dropbox.com/s/zm7a4s6nqelfnso/network_data_early_active_late_con_all_subset_percent_only_vis_bivariatetests.html?dl=1.

An interactive viewer of interaction network in **Figure 7B** is available at https://www.dropbox.com/s/yt8sf6uwhtte55y/network_data_late_active_late_con_all_subset_percent_only_vis_bivariatetests.html?dl=1.

An interactive viewer of interaction network in **Figure 7C** is available at https://www.dropbox.com/s/50tfhif6eqz3uoa/network_data_active_all_subset_percent_only_icu_regression_vis_bivariatetests.html?dl=1.

An interactive viewer of interaction network in **Figure 7D** is available at https://www.dropbox.com/s/q43bz8s294bwobu/network_data_con_all_subset_percent_only_icu_regression_vis_bivariatetests.html?dl=1.

An interactive viewer of cytokine data correlation with mass cytometry data (**Figure 6C**) is available at https://www.dropbox.com/s/6y6bo7zl40qlm26/luminex_correlation_analysis_active_convalescence_group_active_results_vis_stats_results_viewer.html?dl=1.

An interactive viewer of cytokine data correlation with mass cytometry data at convalescence phase (**Figure 6C**) is available at https://www.dropbox.com/s/2h0awk6l4rgzmn9/luminex_correlation_analysis_active_convalescence_group_convalescence_results_vis_stats_results_viewer.html?dl=1.

## Results

### Study Design and Clinical Characteristics of the COVID-19 Cohort

In this study, convalescent patients with different previous clinical trajectories were recruited to aid in the understanding of the COVID-19 immune landscape. To determine the changes in PBMCs induced by SARS-CoV-2 infection in active and convalescent patients, we used a comprehensive mass cytometry panel of 40 antibodies covering lineage-specific markers, adhesion molecules and other surface molecules indicative of the functional state of the cells (**Figure 1A**). This allowed us to study distinct immune cell populations such as T cells, B cells, NK cells, DCs, Mono, basophils, MAIT cells, ILCs and LD Neu, as well as lesser-known immune cell populations such as CD56^+^ Mono, CD56^+/-^ MAIT and the PD-L1^+^ LD Neu immunotypes (21). Cytokine bead arrays based on Luminex™ and ultra-sensitive Quanterix™ technologies were also used to quantify the changes of 28 cytokines in patient plasma (**Figure 1A**). These data were then subjected to network analysis to determine molecular immune signatures of the anti-SARS-CoV-2 response that may drive severe COVID-19 and from those that persist in recovering patients.

Earlier studies by other groups have shown that COVID-19 is a phasic disease (22–24). Here, 87 blood samples were collected from 77 hospitalized COVID-19 patients at varying time points, up to late convalescence and 10 healthy control donors (**Figure 1B**). The state of infection was determined by SARS-CoV-2 real-time reverse transcriptase polymerase chain reaction (PCR) as previously described (16, 25). PCR positive samples were grouped into early or late active, and PCR negative were grouped into early or late convalescence (**Figure 1B**). As a proxy for disease severity, the samples were stratified into three severity groups; mild (symptomatic without supplemental oxygen; n = 32), moderate (symptomatic with supplemental oxygen (suppl. O_2_); n = 24) and severe (suppl. O_2_ and need for intensive care unit (ICU); n = 31) based on the treatment regime. With the exception of age, most demographic variables including gender (χ2 test, 0.0814) did not differ significantly between COVID-19 patients and healthy donors, as well as between severity groups (**Supplementary Table 1**). The association between severity and age was statistically significant (p < 0.0001).

### Temporal Variations in PBMCs during Active and Convalescent COVID-19

To understand the dynamics of circulatory immune cells due to COVID-19 infection, we obtained 38 non-overlapping basic immunotypes from the adaptive and innate branches of the immune system (**Figure 2A** and **Supplementary Table 2**). The abundance of each immunotype was depicted in a bubble plot (**Figure 2B** and **Supplementary Figure 1**). Surprisingly, large numbers of LD Neu were detected in COVID-19 samples, which are also absent in healthy donors (**Supplementary Figure 1**). A targeted mass cytometry panel confirmed that these cells are indeed LD Neu expressing canonical CD66b^+^CD15^+^CD16^high^CD10^+^CD24^+^ phenotype (**Supplementary Table 2**). Also, generalized lymphopenia was observed in active COVID-19 relative to convalescent phase (**Figure 2A**), particularly in various CD4^+^ and CD8^+^ T cell, NKT, MAIT cell and certain B cell immunotypes. We also observed a significant loss of non-classical monocytes (NC. Mono), while increased frequencies were found for plasmablasts, classical monocytes (C. Mono) and LD Neu. Temporal analysis of these immunotypes into four active and convalescent phases further showed their redistribution with disease progression (**Figure 2B**).

We next performed a heatmap analysis to map the temporal changes of immune cells (**Figure 3A**). The earliest responders were elevated levels of NK cells followed by plasmablasts, C./Intermediate (Int.) Mono and LD Neu. In contrary, a sharp decrease in frequency may indicate the mobilization to the inflamed tissues, involving immune cells such as NC. Mono, T regulatory (Treg), T follicular helper (T_FH_), CD4^+^ T and MAIT cells until the late active phase. In particular, the loss in blood MAIT cell frequency was restored to healthy levels during convalescence, supporting its recruitment to the lung mucosa during active infection (26). Also, the expansion of circulating plasmablasts during the active phase is consistent with the single-cell analysis of plasmablasts in severe COVID-19 patients leading to emergency granulopoiesis of neutrophils (27). In contrast, non-class-switched memory (NSM), class-switched memory (CSM) and IgM^+^ memory B cells showed decreased frequencies associated with elevated levels of IL6 during cytokine storm (28). These three memory B cell sub-populations increased during convalescence but were still lower compared to healthy donors (**Figure 3A**).

We then defined various activation and differentiation states of the 38 basic immune cells based on surface marker expression curated in the CellMarker database (**Figure 3A** and **Supplementary Table 2**) (29). Despite the highly heterogeneous changes in immune responses, we still observed some high-confidence trends. CD38, an activation and endothelial-adhesion marker (30, 31), was upregulated across diverse immune cells during active infection, indicative of widespread anti-viral responses (**Supplementary Figure 2**). CD169, an adhesin that binds sialic acid, was also selectively upregulated in all monocytes during the early but not late active phase (**Supplementary Figure 2**). Many T and B cell immunotypes – Treg TEM, NSM and CSM cells – downregulated the lymph node homing molecule CCR6 over the course of disease primarily during active infection (**Supplementary Figure 2**), which may impede homing to inflamed tissues and development of germinal center responses. Additionally, a range of immune cells exhibited elevated levels of CD57 from early active infection to early convalescence, indicative of immune senescence (**Supplementary Figure 2**). Finally, CD16 (or FcγRIII) which recognizes soluble antigen-antibody complexes (32), was upregulated on MAIT cells in late active infection, and on LD Neu, all monocytes and CD16^+^ NK cells during convalescence (**Figure 3A** and **Supplementary Figure 2**).

Based on the major phenotype variations from 38 basic immune cells, we further derived more than 5000 immunotypes to obtain 327 partly overlapping immunotypes (**Figure 1A** and **Supplementary Table 3**). During early active SARS-CoV-2 infection, a transient increased frequency of CD169^+^ C. Mono, CD38^high^ plasmacytoid dendritic cell (pDC) and IgA^+^ plasmablasts were observed. CD141^+^HLADR^-^ C. Mono, CD56^+^ C. Mono, CD45RA^-^ Int. Mono and CD56^+^ Int. Mono peaked during late active infection (**Figure 3B**). We also observed an increase in LD Neu population expressing PD-L1 and CD5 markers from early to late active infection but decline in late convalescence (**Figure 3B**). In contrast, lymphopenia affected virtually all CD8^+^ central (CD8 TCM) and effector memory T cells (CD8 TEM) during active infection, and subsequent T cell subsets recovering to either baseline (ICOS^+^ T_FH_ and HLADR^+^CD38^-^ CD4^+^ T cells) or enriched (Treg TEM and HLADR^+^CD38^+^ CD8^+^ T cells) during late convalescence (**Figure 3B**). Other significant losses included CD45RO^-^ NC. Mono, CD56^+^CD8^+^ MAIT, and CD57^-^CD8^+^ NKT cells during active infection (**Figure 3B**). These are in line with previous studies of preferential T cell lymphopenia, neutrophilia and monocytosis during COVID-19 infection (6, 23, 27, 33, 34). During the entire convalescent period, two groups of immunotypes (“late-con”) did not recover to healthy levels (**Figure 3B**). The first group comprised of frequencies of immune cells higher above healthy levels. These are T cells (such as CD57^+^CD4^+^ T, HLA-DR^+^CD38^+^ CD8^+^ T, CD38^+^/ICOS^+^ T_FH_ defined as circulatory CXCR5^+^, Treg TCM/TEM), NC. Mono (IgA^+^, CD45RO^-^ and CD16^+^CD169^+^) and CCR6^-^ CSM (**Figure 3B**, left). On the other hand, the second group consisted of persistent loss of CD5^-^ conventional type 2 DC (cDC2), Vδ2 TCM, Vδ2 TEM, B cells memory CXCR5^+^, CCR6^+^ CSM and CD5^-^ transitional B cells, even into late convalescence below healthy levels (**Figure 3B**, left). These late-convalescent immunotypes may contribute to post-COVID-19 aberrations. These results indicate dynamic changes to both innate and adaptive cell types among COVID-19 patients ranging from early transient, late active and late convalescent immunotypes.

### Alterations of Immunotypes Associated With Disease Severity

To gain an insight on the impact of severity among active and convalescent patients, we further stratified group I, II and III denoting active mild, active moderate and active severe, respectively, and group IV (severe), V (moderate), and VI (mild) for patients in the convalescent phase, and summarized the frequency changes to key immunotypes in **Supplementary Figure 3**.

**Figure 4:**
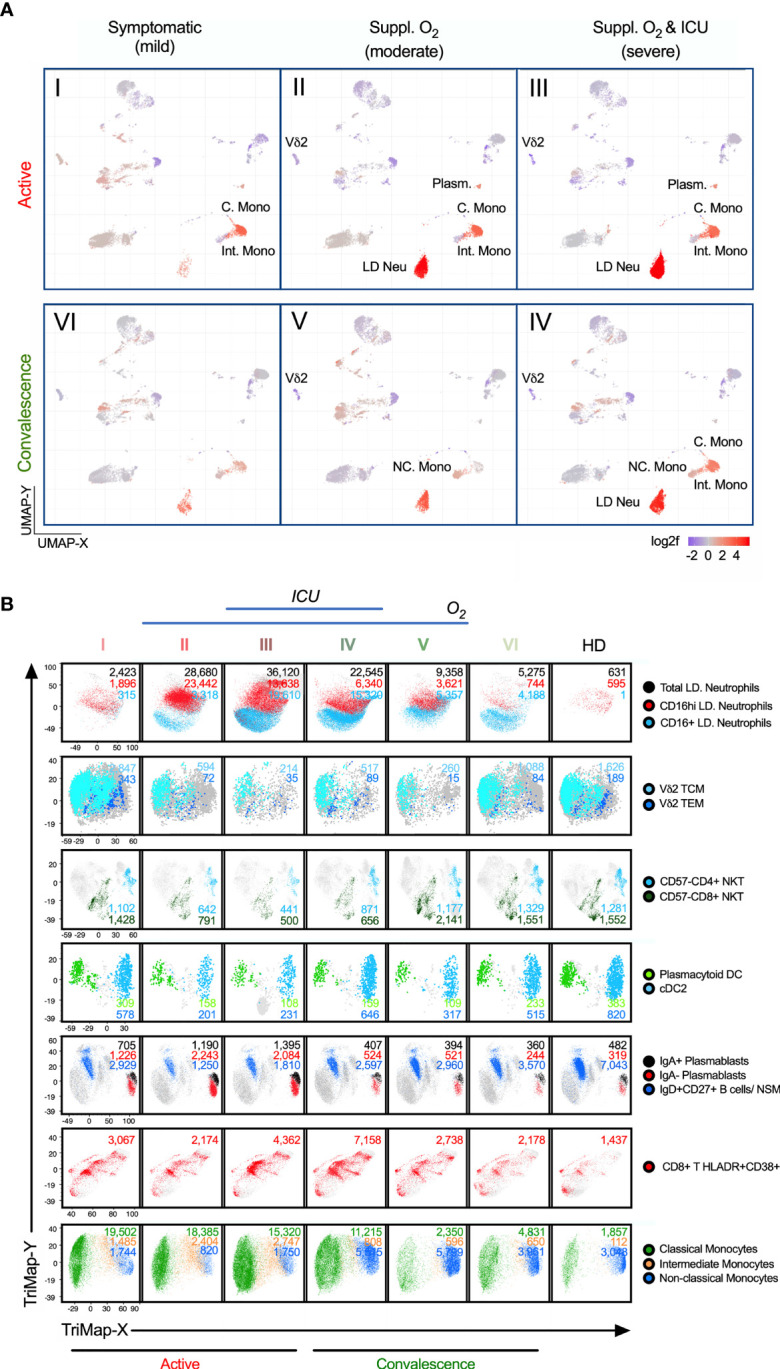
Alterations of immunotypes associated with the six-group disease severity states. **(A)** Distribution of 38 immune cells among group I active mild symptomatic, group II active suppl. O_2_ group III active suppl. O_2_ ICU, group IV convalescent suppl. O_2_ ICU, group V convalescent Suppl. O_2_ and group VI convalescent mild symptomatic using UMAP clustering. Color indicates the log2 fold change in the frequency against healthy donors. **(B)** TriMap clustering of CD16^+/hi^ LD Neu, Vδ2 TCM, Vδ2 TEM, pan-CD57^-^ NKT, pDC, cDC2, IgA^+^/^-^ plasmablasts, IgD^+^CD27^+^ NSM, HLA-DR^+^CD38^+^ CD8 T cells, C. Mono, Int. Mono and NC. Mono among 6 groups of SAR-CoV-2 patients and healthy donors (HD). The absolute number shown for the immune cells has been normalized per 300,000 PBMCs and thus reflects its frequency.

The most striking observation was the high levels of severity-associated LD Neu and high LD Neu-to-lymphocyte ratios (33), even in convalescent individuals who had experienced moderate to severe COVID-19 (**Figure 4A** and **Supplementary Figure 4A**). The expansion of LD Neu fraction was accompanied by an increase of CD16^+/high^ neutrophils. CD16^high^ neutrophils (**Figure 4B** and **Supplementary Figures 4B, C**) described herein as pseudo-Pelger-Huet cells were previously reported in other severe viral infections (33).

Among the T cells, we found the strongest association with severity to be the HLADR^+^CD38^+^ CD8^+^ T cells. Unlike the largely invariant frequencies of MAIT and NK cells, which remained low across groups I to V (**Supplementary Figures 5A, B**), hyperactivated HLADR^+^CD38^+^ CD8^+^ T cells was more pronounced in groups III/IV, in particular group IV (**Figure 4B** and **Supplementary Figure 6A**). This is in line with previous report on CD8^+^ T cells co-expressing CD38 and HLA-DR in hospitalized patients by Matthew et al. (34). In this study, we further showed that these CD8 T cells continue to persist in convalescent severity. Also, many studies have suggested a role of inflammatory monocytes in the pathogenesis of COVID-19 (9, 35, 36). Among the monocytes affected by COVID-19, CD14^+^CD16^-^ C. Mono and CD14^+^CD16^+^ Int. Mono accounted for 91-96% of total monocytes, which remained high in convalescent severe patients (68%) relative to healthy donors (39%) (**Supplementary Figure 4D**), which is in agreement with Zhou et al. (36). Notably, there is a higher fraction of Int. Mono with more severe COVID-19 cases in blood monocytes, relative to convalescent and healthy donors (**Supplementary Figure 4D**). Also, the loss of CD14^low^CD16^+/++^ NC. Mono during COVID-19 prominently increased in groups IV/V, relative to mild group VI and healthy donors (**Supplementary Figure 4D**). More interestingly, we observed an injury-related switch in the monocytes (37, 38), which enriched NC. Mono frequency in the convalescent blood (2% in both groups IV/V) despite a much smaller monocyte fraction in group IV (32%) than V (66%). (**Figure 4B** and **Supplementary Figure 4D**). We further analyzed the phenotypic alterations of the monocytes. Here, increased CD169 expression across the monocytes is disease- and not severity-related (**Supplementary Figures 4E, F**). More interestingly, more NC. Mono expressing CD86 and CD123 markers were found in convalescent severe individuals, which persisted till late convalescence (**Supplementary Figures 4E, F**).

Two other severity-associated immunotypes are Vδ2 T and NKT cells (**Figure 4B** and **Supplementary Figure 7**). Their frequencies sharply declined in groups II/III/IV patients. The losses of Vδ2 T are mostly Vδ2 TCM and Vδ2 TEM (**Figure 4B** and **Supplementary Figure 7A**). Similarly for NKT cells reported by Zingaropoli et al. (39), we further showed that all CD57^-^ immunotypes (CD4^+^, CD8^+^ and DN) but not the senescent CD57^+^ NKT cells, was markedly reduced in groups II/III/IV (**Supplementary Figure 7D**). Instead, CD57^+^ NKT cells were slightly expanded in groups IV/V (**Supplementary Figure 7D**). Other affected immunotypes, which remained low in convalescent severe individuals included dendritic cells; pDC and cDCs. (**Figure 4B** and **Supplementary Figures 7B, C**). As for the plasmablasts, its expansion during the active phase recovered to near healthy levels, and thus did not associate with convalescent severity (**Figure 4B** and **Supplementary Figure 8**). The results taken together indicate that there were increased and decreased frequencies in specific immunotypes associated with disease severity.

### Clustering Analysis of Immunotypes Based on Disease Severity

**Figure 5:**
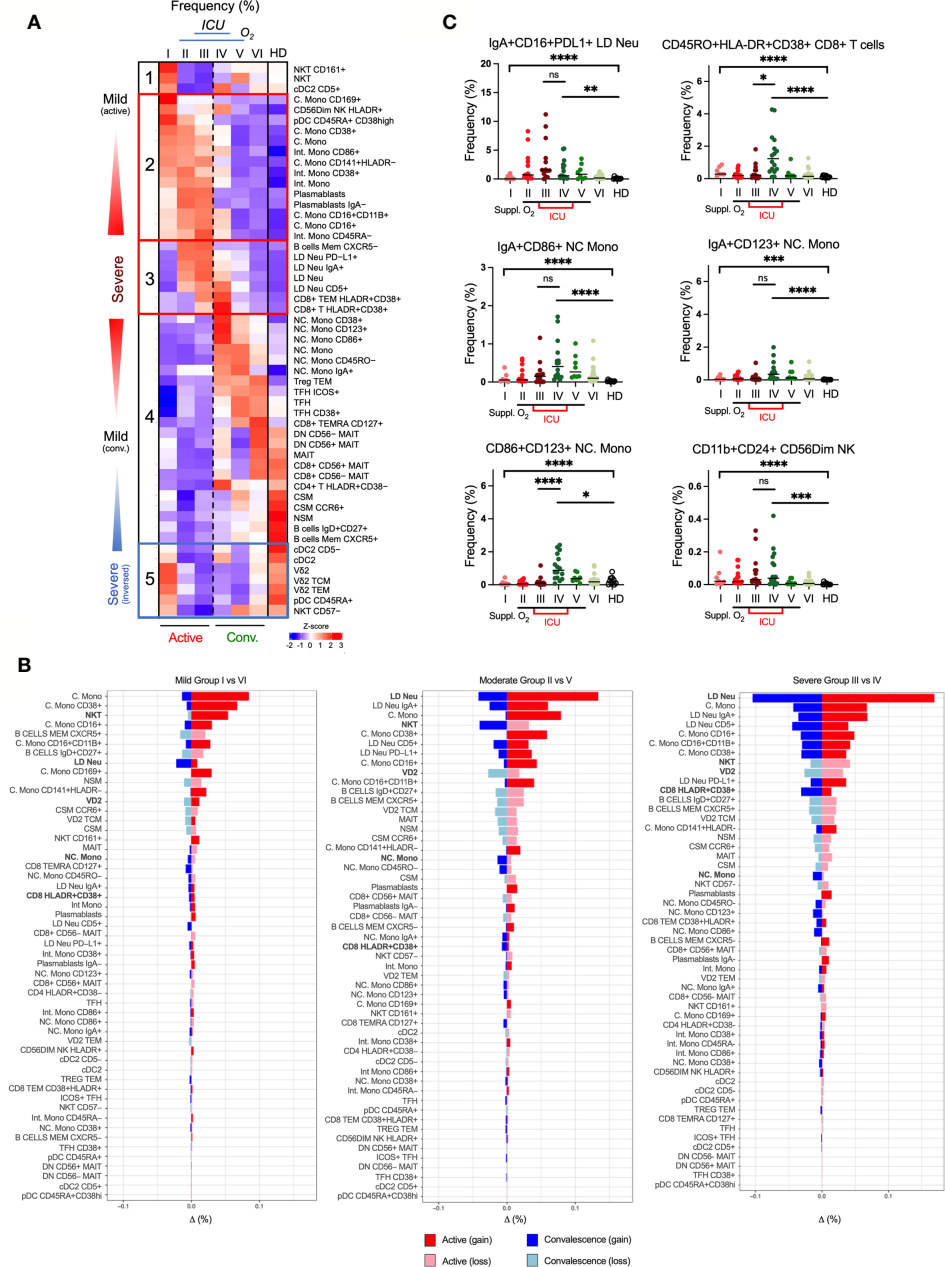
Association of immunotypes with COVID-19 disease severity in active and convalescent individuals. **(A)** Left: Heatmap of CyTOF data of frequencies of 53 immune cell populations among the 6 group severity stratifications and divided into five clusters. Right: Box-and-whiskers plots showing means and IQR increased and reduced frequency of immune cell pollutions with disease severity. **(B)** Enumeration of immune cell frequencies compared across severity groups against healthy donors. Selected immunotypes mentioned in this study are shown in bold. **(C)** Profiles of immunotypes persisting in convalescent severe patients. Immunotypes of LD Neu, HLA-DR^+^CD38^+^ CD8^+^ T cells, CD86^+^/CD123^+^ NC. Mono and C56^Dim^ NK cells, are further defined by co-expression of PD-L1, IgA, CD11b, CD16, CD24 or CD45RO frequencies. Scatter plots depict the means with SEM. ns, not significant, *p < 0.03; **p < 0.002; ***p < 0.0002, ****p < 0.0001 (Kruskal-Wallis test with multiple comparison corrected on each disease severity group versus total healthy). See **Supplementary Figure 7** for comparisons with Vδ2 T and NKT cells.

To determine the relative contribution of each immunotype in promoting disease severity, we analyzed the extended immunotypes with disease severity (**Figures 5A, B**). A group of five clusters based on the immune responses and disease severity was observed (**Figure 5A**). The first cluster comprised of CD161^+^ NKT and CD5^+^ cDC2 increasing in the mild group I, suggesting a disease-protecting role. The second cluster is a transitional severe group positive association across the 3 active groups. These included high levels of plasmablasts, CD169^+^/CD38^+^ C. Mono and CD86^+^/CD45RA^-^/CD38^+^ Int. Mono but not total monocytes. Moreover, increased HLA-DR^Low^CD141^+^ C. Mono, which was previously reported in ICU patients displayed equivalent changes across our active mild and severe COVID-19 (8).The third cluster was positively associated with severe COVID-19 infection showing increased CXCR5^+^ memory B cells in groups II/III, and many LD Neu immunotypes such as IgA^+^, PD-L1^+^ and CD5^+^ in ICU groups III/IV (**Figures 5A–C**). Here, hyperactivated HLA-DR^+^CD38^+^ CD8^+^ memory T cells significantly increased in ICU groups III/IV (**Figures 5B, C**). The fourth cluster exhibited disproportionate frequency changes during convalescence. Various Treg TEM, T_FH_ and NC. Mono immunotypes (CD38^+^/CD123^+^/CD86^+^/CD45RO^-^/IgA^+^) expanded across groups III/IV/V/VI above healthy levels (**Figures 5A–C**). For example, IgA^+^ NC. Mono immunotypes were found elevated in both ICU groups III/IV but CD86^+^CD123^+^ NC Mono significantly increased only in convalescent severity group IV (**Figures 5B, C**). On the other hand, immunotypes of MAIT, CSM and NSM of more severe COVID-19 patients remained below healthy levels (**Figure 5A**). Finally, the fifth cluster defined as “severe inverse” showing a decline in the frequencies with increased disease severity. These immunotypes included cDC2, CD45RA^+^ pDC, CD57^-^ NKT and Vδ2 memory T cells (**Figure 5A**). Of note, the frequency of transdifferentiating CD56^dim^NK expressing CD11b^+^ and CD24^+^ markers was found to be increased in ICU groups III/IV (**Figure 5C**), and remained low even in late convalescence (40). These results indicate the contribution of distinct immunotypes in the trajectory of COVID19 symptoms.

### Dynamics of Plasma Cytokine Levels in COVID-19 Patients

**Figure 6:**
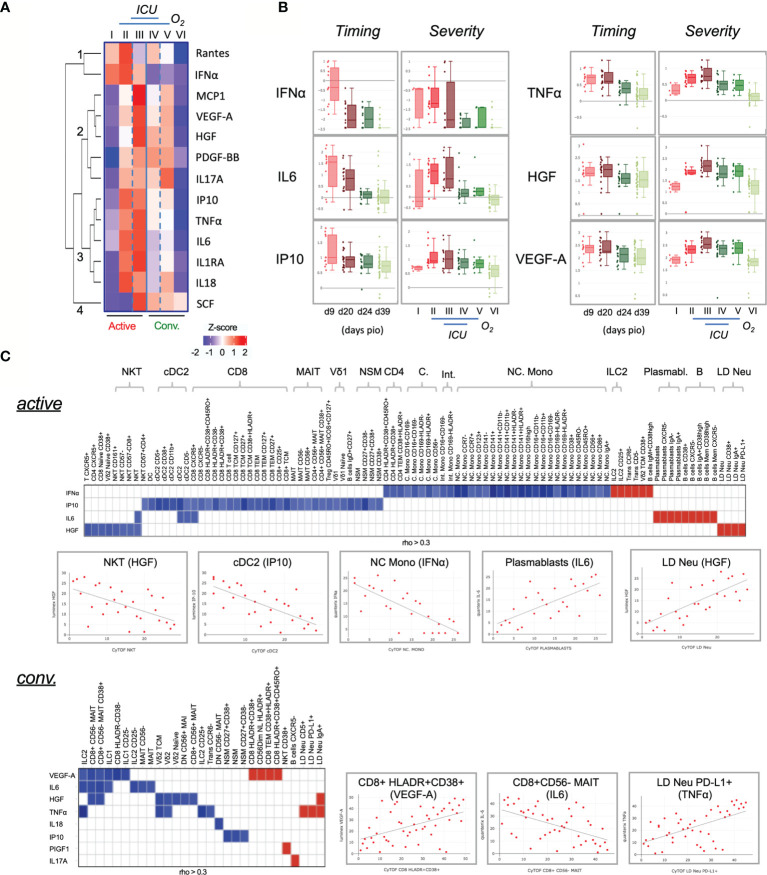
Characterization of cytokines in COVID-19 patients. **(A)** Changes in cytokine levels among COVID-19 patients based on timing and severity. The heatmap shows the z-scores of the mean logarithmically transformed concentration of the 13 cytokines (of a total of 28) showing significant differences between any of the 6 severity groups. The z-scores are colored in red for positive values and in blue for negative values. The cytokines are clustered using hierarchical clustering using Euclidean distances into four clusters, which are labeled 1 to 4 in the figure. **(B)** Box plots of selected cytokines showing differences in timing and/or severity. The timing (left panels) refers to the plasma cytokine levels detected on the respective day post illness onset (PIO), the severity (right panels) to the levels detected in the 6 severity groups. Red colors refer to samples from the active phase, green to convalescence phase. An interactive viewer is available in the online content: data availability section. **(C)** Associations between cytokine level and cell frequency during active and convalescent phase. The heatmap displays the strength of the association indicated by the correlation coefficient (rho). Color indicated the direction (Red: positive, blue: negative). Only associations abs(rho) > 0.3 and p < 0.05 are shown. Selected examples of these correlations are shown in the scatter plots. An interactive viewer is available in the *Materials and Methods: Data and Code Availability* section.

In COVID-19 patients with severe disease, cytokine storm causes uncontrolled inflammatory responses by own immune system that can lead to death (10, 41). We next employed either Luminex or high-sensitivity Quanterix bead arrays to identify persistent cytokine production in COVID-19 patients. Using the same groups of COVID-19 patients, 13 out of 28 cytokines showed statistically significant associations with the six severity groups (adjusted p < 0.05) (**Figure 6A**). Our analysis revealed that IFNα, while abundantly found in the early active phase, did not indicate severity (**Figure 6B**). Also, increased IL6 is known to worsen disease and reduce T cells (42), but only increased in the active phase (**Figure 6B**). The association with severity however substantially improved for IP10, TNFα, HGF and VEGF-A. Especially the latter two remained at high levels in both the active and convalescent severity phases (**Figure 6B**).

To further understand the abnormal levels of cytokines, various correlative plots with 327 immunotypes are provided in an interactive online viewer (see *Materials and Methods: Data and Code Availability*). The natures of immune responses are shown in **Figure 6C**. During active infection, IFNα, IP10, IL6 and HGF showed a pleiotropic effect on several immune cells (**Figure 6C**, top). IFNα had a negative correlation with various monocytes such as NC. Mono but positively associated with ILC2, CD38^+^ Vδ2 TCM, IgM^+^CD38^high^ B cells, CD5^-^ transitional B cells, CCR6^-^ transitional B cells and CD25^+^ ILCs. IL6 was positively associated with B cells and various plasmablasts, and HGF was negatively associated with NKT but positively with LD Neu. During convalescence, a greater number of cytokines but fewer associations were found with various immunotypes than in the active phase (**Figure 6C**, bottom). Interestingly, IgA^+^, CD5^+^ and PD-L1^+^ LD Neu positively associated with TNFα, which is known to promote neutrophil degranulation (43, 44). Additionally, PIGF1 and IL17A positively associated with CD38^+^ NKT and CXCR5^-^ B cells, respectively. And negative associations of both proinflammatory TNFα and HGF with Vδ2 T cells were observed, as well as negative associations of both IL6 and HGF with CD8^+^CD56^-^MAIT (**Figure 6C**, bottom). Here, VEGF-A negatively associated with CD8^+^CD56^-^ MAIT cells but was strongly associated with HLA-DR^+^CD38^+^ CD8^+^ T cells during convalescence (**Figure 6C**, bottom).

### Network Analysis of Immunotypes and Plasma Cytokines

To determine the immunopathogenesis of COVID-19, we integrated the plasma cytokines, immunotypes, and clinical symptoms into a database and performed Bayesian network analysis.

**Figure 7:**
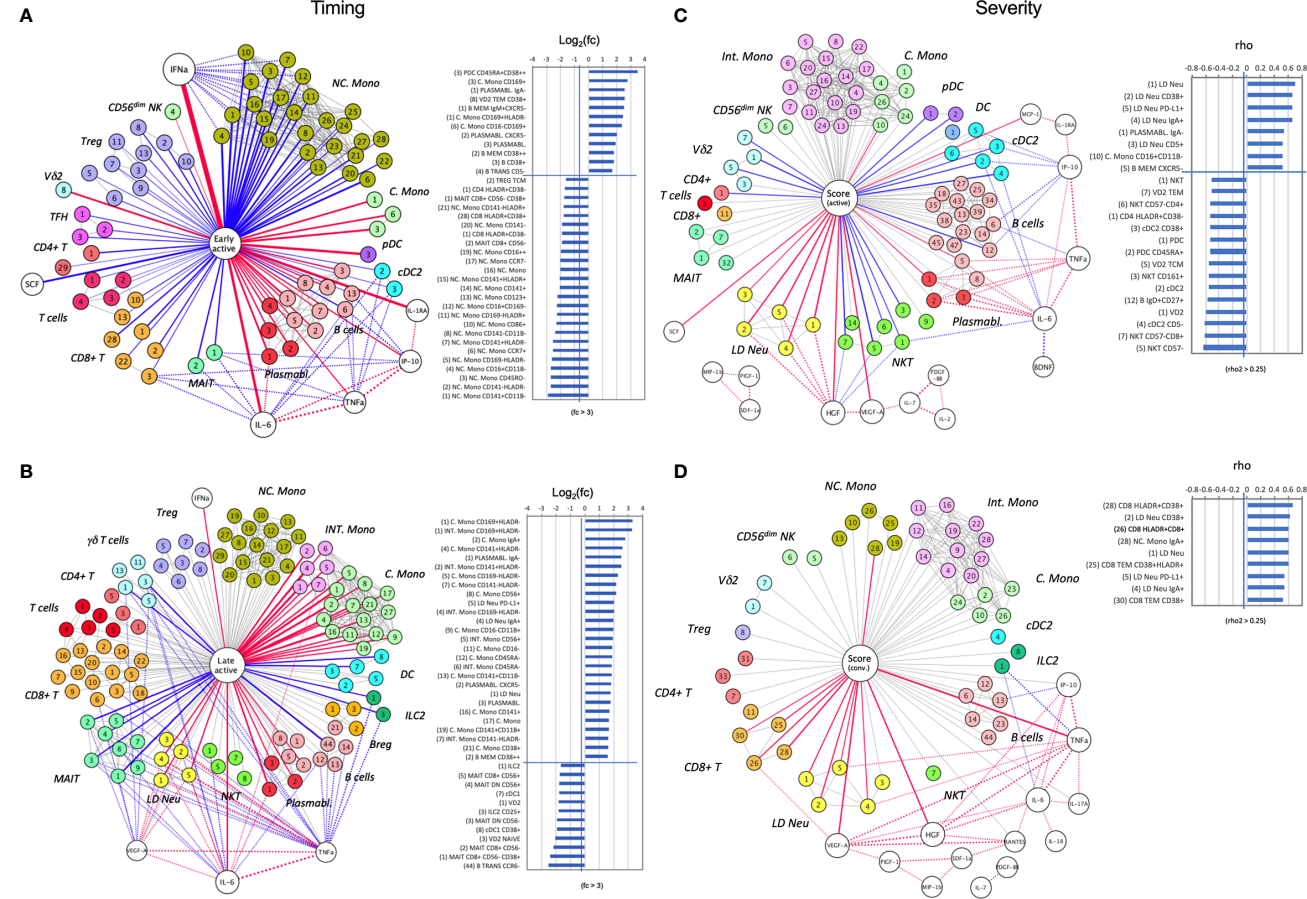
A node-edge interaction network of the cytokine level and immune cellular frequencies in COVID-19 patients. Association are shown with regard to the timing **(A, B)** and the severity **(C, D)**. Nodes represent either cytokines (white) or immunotypes (colored). The central node represents the “comparison of interest”. The edges represent significant associations between two nodes with the thickness indicating the strength either based on fold change or correlation coefficient (rho). Color indicates the direction (Red: positive, blue: negative), dotted lines indicate associations with cytokines. For the central node, only associations with abs(rho) > 0.3 and p < 0.05 are colored and shown as bar charts on the right. For the timing **(A, B)** these bar charts indicate the fold changes in the early active; median day 9 PIO **(A)**, and late active state; median day 24 PIO **(B)** in reference to late convalescent state while for the severity **(C, D)** they represent the correlation coefficient (rho) in reference to the severity groups in the active **(C)** and convalescent state **(D)**. The number code of the immunotype is listed in **Supplementary Table 3**, an interactive network viewer is available in the *Materials and Methods: Data and Code Availability* section. ^++^ denotes highly stained immunotype.

In the early active phase of SARS-CoV-2 infection, a dynamic range of anti-viral responses was observed. Hierarchical positive correlations were observed for IFNα, followed by IL6 and IL1RA (**Figure 7A**). Notably, elevated levels of IFNα inversely correlated with a general depletion of NC. Mono populations, including the CD141^+^CD11b^-^, CD141^-^HLA-DR1^-^, CD45RO^-^ and CD16^+^CD11b^-^ immunotypes (fold change > 3) (**Figure 7A**). On the other hand, higher levels of early responders are CD38^high^ CD45RA^+^ pDC, activated B memory, VδVmT cells, plasmablasts, and C. Mono immunotype expressing a CD169 marker directly induced by IFNα (**Figure 7A**). Of these, IgA^-^ plasmabasts positively correlated with plasma IL6. In parallel, this increased in IL6 was associated with a loss of CD8^+^ T cells, MAIT and cDC2 (**Figure 7A**). In addition, elevated IP10 was associated with a depletion of MAIT and certain B cell immunotypes (NSM CD27^+^CD38^-^ and NSM CD27^-^CD38^+^) (**Figure 7A**). We did not find any association of SCF and IL1RA with any immunotypes (**Figure 7A**).

With disease progression, the dominating IFNα levels as observed in the early phase waned (**Figure 7B**). Although the correlation with NC. Mono nearly diminished, Int. and C. Mono strongly expanded in this phase, in particular for the CD169^+^ immunotype with a 8-fold increase in frequency (**Figure 7B**). Also, the earlier correlations between MAIT, IgA^-^ plasmablasts and IL6 levels maintained at the late active phase. Next, two other cytokines, VEGF-A and TNFα, begun to show multiple cellular associations with significant depletion of γδ T, ILC2 and MAIT cells, while IL6 was associated with late increase in LD Neu and Int. Mono (**Figure 7B**). These correlations potentially link different cytokines to lymphopenia, neutrophilia and monocytosis, which are characteristic of SARS-CoV-2 infection.

A strikingly different picture emerged when the network analysis was performed for severe COVID-19 immune responses found in ICU patients. During the active phase, the strongest positive correlation was observed for increased levels of HGF and severity-related LD Neu and its CD38^+^, PD-L1^+^, IgA^+^ and CD5^+^ immunotypes (**Figure 7C**). On the contrary, increase in HGF levels was associated with a loss of NKT cells, namely the CD57^-^ immunotype, which may be a consequence of their redistribution from peripheral blood to the inflamed tissue. Other immunotypes, which were positively associated with severity included IgA^-^ plasmablasts and CD16^+^CD11b^-^ C. Mono (**Figure 7C**), possibly pointing to the role of IgG in COVID-19 pathogenesis. Moreover, during active severe COVID-19, increased plasmablast frequency directly correlated with IL6 level, which was instead inversely associated with cDC2 (**Figure 7C**). Similarly, other inverse associations with active severe COVID-19 included pDC and Vδp T cells in the blood (**Figure 7C**), which suggests the propensity of these immune cells to home to the lungs or other inflamed tissues during active severe COVID-19. For convalescent ICU cases, there was no inverse association found in the blood (**Figure 7D**). Instead TNFα, HGF and VEGF-A correlated directly with convalescent severity. The increase in TNFα but not HGF was positively associated with IgA^+^ LD Neu and PD-L1^+^ LD Neu (**Figure 7D**) (44). Elevated VEGF-A level was strongly associated with enriched HLA-DR^+^CD38^+^CD8^+^ T cells (**Figure 7D**), which can drive CD8^+^ T cell exhaustion in tumors (45). On the other hand, only IgA^+^ NC. Mono was strongly associated with convalescent severity (**Figure 7D**), based on elevated frequency in ICU groups III/IV (**Figure 5C**). Finally, IFNα and IL6 served only as early immune responses directed against SARS-CoV-2 (**Figures 7A, B** and **Supplementary Figure 9**) but not the severity of the disease in both active and convalescent COVID-19 phases.

## Discussion

Much work has identified the immunological and inflammatory profiles of COVID-19 patients with severe disease but little is known about the convalescent patients. In this study, we show that individuals recovering from severe COVID-19 present persistent features of immunopathology. These are mainly caused by collateral or even autoimmune-like damage inflicted by a hyperactivated immune system. Presently, about 2.5% convalescent COVID-19 patients develop thrombosis at 30 days post discharge, or mucormycosis which can overburden the healthcare (46, 47).

Convalescent severe patients recovering from COVID-19 lung damage showed persistent loss or abundance of immune cells that can contribute to host protection or disease progression. These include reduction of NKT (e.g. CD57^-^) and Vδ2 (e.g. TCM) in blood, which contributed to viral clearance in lungs (7, 39, 48), and possible signs of inflammation (49). On the other hand, we found major increased of LD Neu such as PD-L1^+^ in severe COVID-19, that can suppress cytotoxic T cells (9, 50, 51). Degranulation is an integral part of neutrophil biology hence the number of LD Neu in the blood may indicate ongoing neutrophil responses. Previous COVID-19 studies have reported abundant activated neutrophils inside the inflamed lung tissue (52–55), and higher levels of immature neutrophils in blood (7). Notably, LD Neu have a higher capacity to release NETs (56), which were found in the lungs of deceased COVID-19 patients (57). Interestingly, LD Neu and NET formation have been reported in several autoimmune diseases, such as antiphospholipid syndrome (58), systemic lupus erythematosus (59), and anti-neutrophil cytoplasm autoantibody vasculitis (60). Thus, excessive circulating neutrophils can contribute to lung injuries (61), and complicate recovery among convalescent patients.

Monocytes have been implicated in COVID-19 pathology with contrasting results in decreased or increased levels of C., Int. and NC. monocytes (8, 62–64). Interestingly, we found that LD Neu can contaminate these monocytes during cytometry data analysis, and performed UMAP clustering to first segregate the basic immunotypes. Hence we have characterized about 90 different monocytes distinct from the LD Neu population to identify key contributors. As expected, IFNα induced CD169^+^ C. Mono during early phase, as well as increased CD16^hi^ monocyte populations. However, we did not observe any disease-severity associations. Here, we also did not observe increased CD141^+^HLADR^-^ C. Mono in severe cases as reported by Hadjadj et al. (65). A possible explanation may be attributed to the sample time-point or definition of severity in Hadjadj’s study; ‘mild’ cases were asymptomatic but our patients were all hospitalized. Instead we found an expansion of inflammatory CD16^+^ monocytes, CD16^+^CD14^+^ Int. Mono and CD16^+^CD14^-^ NC. Mono. Of note, the expansion of pro-inflammatory CD16^+^ monocytes can produce cytokines TNF-α, IL6 and IL10 in response to microbial-associated molecular patterns (66, 67).

Among the CD16^+^ monocytes, Int. Mono is responsible for T cell proliferation stimulation (68), and can augment IL6 inflammation leading to severe COVID-19 lung damage (36). Indeed abundant Int. Mono was found in severe COVID patients, which subsequently declined in convalescence. On the other hand, NC. Mono are patrolling monocytes in the vasculature (69), and remain elevated in convalescent severe blood. Their other abilities include antigen presentation, T cell stimulation and even neutrophil recruitment to vascular endothelial cells (68, 70, 71). The high level of NC. Mono is attributed to the switching of C. Mono directly to NC. Mono and macrophages at inflamed sites (37, 38). These patrolling NC Mono are custodians of vasculature (72), and thus promote healing of inflamed tissues (73). However, we found a distinct co-stimulatory CD86^+^CD123^+^ NC. Mono subset elevated in convalescent severe cases. The increased CD86^+^ expression is likely through IFN-γ stimulation, and is associated with high antigen presentation (74, 75). Notably, CD86^high^ NC. Mono may drive pathogenic CD4^+^ T cell polarization and Ig production, as reported in system lupus erythematosus and chronic Chagas disease (76, 77). Additionally, healthy monocytes normally have a low expression of CD123, and its aberrations are found in various hematologic malignancies (78, 79). Thus, accumulating CD86^+^CD123^+^ NC. Mono levels may contribute to ongoing inflammatory responses.

Unlike phagocytic C. Mono expressing CCR2^+^CX3CR1^low^, pro-inflammatory NC. Mono are exclusively CCR2^-^CX3CR1^high^ (80), and thus migrate to different ligands CCL2 and CX3CL1, respectively. Presently, KAND567 an inhibitor targeting CX3CR1 is in clinical trial for treating COVID-19 hyper-inflammation (81). More recently, elevated NC. Mono was reported to persist up to 16 months post infection among post-acute sequelae SARS-CoV-2 or long-COVID-19 patients in the presence of TNF (70, 82). Indeed we observed higher levels of TNF-α that can promote the survival of NC. Mono found in convalescent severe patients. The same trend was observed within the IgA immune system, IgA is the dominant antibody isotype ascribed to early novel SARS-CoV-2 infection (83, 84). In this work, high level of IgA deposited on LD Neu and NC. Mono are preserved in convalescent severity, and can form circulating IgA-FcαRI complexes (85). Such IgA immune complexes can resolve mucosal infection but in excess can become detrimental (86). Therefore, a single wave of NC. Mono can become wound healing macrophages, initiate and propagate immune responses (87). However, excessive levels of IgA^+^/CD86^+^/CD123^+^ NC. Mono in convalescent severe COVID-19 patients may correspond to incomplete disease resolution.

Another notable feature among convalescent severe patients is the strong T cell activation. A significant increase of peripheral hyperactivated HLA-DR^+^CD38^+^ CD8^+^ T cell expressing CD45RO^+^ memory phenotype in some convalescent severe patients is interesting considering its oblivion during active severe COVID-19. These highly activated CD8^+^ T cells are critical in viral control, and have been found in lungs of deceased COVID-19 patients (2, 88). Such focal hyperactivation of CD8^+^ T cells but not CD4^+^ T cells is similar to those of other acute viral infections and vaccines but were relatively short-lived in peripheral blood (89, 90). The stability of CD8^+^ T cell activation suggested prolonged peak immune responses preserved even in the convalescent period. Moreover, the differentiation into memory-type T cells suggested the durability of T cell response in viral clearance and maintained immunity. Thus, the frequency of HLA-DR^+^CD38^+^ CD8^+^ T cell in convalescent severe patients may suggest a failure to down-regulate responses due to earlier overactive immune responses. Another finding is an unreported frequency of transdifferentiating NK cells that were marginally elevated in severe COVID-19 and convalescent severity. These CD11b^+^CD27^+^ NK cells which express c-Kit, are myeloid progenitor of neutrophils and monocytes (40), and may contribute to the persistent signs of immunomodulatory in convalescent severe COVID-19. Thus, a key question will be how will these elevated immune responses change over time with new COVID-19 variants.

In summary, as COVID-19 continues to plague the nations with only some parts of the world progressing to reopening, the possibility of waves and variants-of-concern outbreaks remain. Identifying early inflammatory immunopathology can minimize a patient’s vulnerability in developing more severe diseases, which can be compounded by comorbidities. Our study revealed characteristics of prolonged overactive state of the immune system. Since activated immune cells are likely to generate cytokines, targeting specific cytokines with inhibitors may calm the immune reactions. Out of the 327 immunotypes, we have obtained the strongest severity associations between LD neutrophils and HGF in active patients, and HLADR^+^CD38^+^ CD8^+^ T cells and VEGF-A in convalescent patients. Notably, there were also elevated NC. Mono, HGF and TNFα levels among convalescent severe patients compared to healthy adults, which are associated with lung injury (1). Thus, our understanding of distinct immunotypes that reflect their clinical feature and disease severity may aid in the management of post-COVID-19 symptoms.

### Limitations

Our study is subject to certain limitations. Although fewer female patients were admitted at the time of the study, the gender imbalance was found not significant (χ2 test, 0.0814). Instead, age remains a variable; The COVID-19 patients had a median age of 52 and were older than HCs’ median age of 34. Additionally, the cytokine levels for healthy donors were unavailable for reference comparison.

## Data Availability Statement

The datasets presented in this study can be found in online repositories. The names of the repository/repositories and accession number(s) can be found in the article/**Supplementary Material**.

## Ethics Statement

The studies involving human participants were reviewed and approved by National Healthcare Group Domain Specific Review Board. The patients/participants provided their written informed consent to participate in this study.

## Author Contributions

Conceptualization: JL, KP, LN, BL, and OR. Methodology: JL, KWT, CYL, KPT, GC, Y-HC, CP, CYPL, S-WF, NK-WY, RS-LC, SA, ZC, MT, AT-R, NL, and WH. Software: KD and BL. Validation: JL and KP. Formal Analysis: JL, KP, LW, KD, BL, OR. Resources: S-YT, SK, DL, Y-SL, SO, and BY. Data Curation: KD and BL. Writing – Original Draft: JL, KP, LW, BL, and OR. Writing – Review & Editing: JL, KP, LW, BL, and OR. Visualization: JL, KP, LW, BL, and OR. Supervision: LR, LN, and OR. Project Administration: LN and OR. Funding Acquisition: LN. All authors contributed to the article and approved the submitted version.

## Funding

This work was supported by A*STAR Infectious Diseases Labs and Singapore Immunology Network (SIgN) core research grants, and the A*STAR COVID-19 Research funding (H/20/04/g1/006) provided to SIgN by the Biomedical Research Council (BMRC). Subject recruitment, sample collection and analyses were funded by the National Medical Research Council (NMRC) COVID-19 Research Fund (COVID19RF-001, COVID19-RF007, COVID190RF-060). The SIgN Immunomonitoring Platform is supported by a BMRC IAF 311006 grant and BMRC transition funds #H16/99/b0/011. The SIgN Flow Cytometry and the Multiple Analyte Platforms were supported by a grant from the National Research Foundation, Immunomonitoring Service Platform ISP) (#NRF2017_SISFP09) and the National Research Foundation Singapore (NRF). The funders had no role in the design and conduct of the study; collection, management, analysis and interpretation of the data; preparation, review, or approval of the manuscript; and decision to submit the manuscript for publication.

## Conflict of Interest

The authors declare that the research was conducted in the absence of any commercial or financial relationships that could be construed as a potential conflict of interest.

## Publisher’s Note

All claims expressed in this article are solely those of the authors and do not necessarily represent those of their affiliated organizations, or those of the publisher, the editors and the reviewers. Any product that may be evaluated in this article, or claim that may be made by its manufacturer, is not guaranteed or endorsed by the publisher.
