# Supplementary material for: Data-Driven Analysis of COVID-19 Reveals Persistent Immune Abnormalities in Convalescent Severe Individuals
Source: Front Immunol. 2021 Nov 19;12:710217. doi: 10.3389/fimmu.2021.710217 (PMC8640498; doi:10.3389/fimmu.2021.710217)
Supplement: Supplementary file 1 [file DataSheet_1.docx]

Supplementary Material

**Supplementary Figure 1.** Bubble plots of immune subset frequencies for active (left subplot) and convalescence (right subplot) COVID-19 patients compared to healthy donors. The size of the bubble shows the percentage of the subset while the bubbles are colored by the log two fold change (red for increase over healthy and blue for decrease over healthy). The bubble positions are based on the median of the UMAP dimensions.

**Supplementary Figure 2.** Temporal changes in surface marker expression profiles of various immunotypes during active and convalescent COVID-19. Top: The profile analysis depicted for each grouping is indicated for each active or convalescent phase. The heat map on the top is colored by the number of significant markers (scored by matrix_1) on the subsets, which are associated with temporal changes for each of the pattern ID. The most frequent surface marker expressions (e.g. CD38 and IgA during active infection) across various immunotypes are shown as darker red. For each pattern ID, ‘1’ denotes up/down-regulation and ‘0’ denotes baseline with respect to healthy levels. Bottom: Box-and-whiskers plots of selected immunotypes and pattern ID showing means, and IQR up-regulation and down-regulation of surface markers associated with early/ late active and early/late convalescent phases. Comparisons were performed between patients groups and late convalescence using Kruskal-Wallis test followed by Dunn’s post hoc tests. Asterisks indicate statistical significance - *, p<0.05; **, p<0.01; ***, p<0.001 (Kruskal-Wallis test with multiple comparison corrected on all disease phases and healthy controls).

**Supplementary Figure 3.** Alterations of immunotypes associated with mild and severe during active and convalescent infection. (A) Left: Heatmap of CyTOF data of frequencies of 38 main immune cell populations among the 6 groups severity stratifications. Right: up- or down-regulation of indicated surface markers for the 38 basic immune cell populations among 6 group severity stratifications. Asterisks indicate statistical significance - *, p<0.05; **, p<0.01; ***, p<0.001 (Kruskal-Wallis test with multiple comparison corrected on all disease phases and healthy controls). (B) Top: The pattern profile of surface markers are binned in accordance to the severity groups I to VI, whereby ‘1’ denotes up/down-regulation and ‘0’ denotes baseline with respect to healthy levels. The heat map on the top is colored by the number of significant markers on the subsets (scored by matrix_1), which are associated with severity for each of the pattern ID. Bottom: Box-and-whiskers plots of selected immunotypes showing means, and IQR up-regulation and down-regulation of surface markers associated with active/mild, active/severe, severe, conv./severe, and conv./mild clinical status. Comparisons were performed between patients groups and late convalescence using Kruskal-Wallis test followed by Dunn’s post hoc tests. Asterisks indicate statistical significance - *, p<0.05; **, p<0.01; ***, p<0.001 (Kruskal-Wallis test with multiple comparison corrected on all disease phases and healthy controls).

**Supplementary Figure 4**. Neutrocytosis and monocytosis during SARS-CoV-2 infection. (A) Neutrocytosis due to SARS-CoV-2 infection is mild among symptomatic group I patients but persisted in severe convalescence group. A higher low-density neutrophil-to-lymphocyte ratio is associated with disease severity. (B) Frequency distribution of low-density neutrophil population whereby the CD16^+^ LD neutrophils are mostly mature neutrophils in healthy donors but co-mixed with immature neutrophils from the CD16^int^ LD neutrophils in COVID-19 patients. The presence of CD16^++/high^ LD neutrophils described as pseudo-Pelger-Huet cells, are absent in healthy donors. (C) The pseudo-coloured plots of LD neutrophil gated as CD16^lo^, left shift CD16^int^, CD16^+^ and CD16^high^ immunotypes and their indicated cell count out of 210K human PBMCs. (D) Monocytosis is apparent even in symptomatic group I and decreases with convalescence, and CD16 marker upregulation in NC. Monocytes. Results show a smaller NC. Mono fraction in group IV still contributed to an overall similar blood frequency as group V. (E) Comparison of NC. monocytes and other monocytes during COVID-19 disease severity. Scatter plots depict the means with SEM. ns: not significant, *, p <0.03; **, p <0.002; ***, p <0.0002, ****, p <0.0001 (Kruskal-Wallis test with multiple comparison corrected on each disease severity group versus total healthy). (F) Frequency changes of representing monocyte immunotypes associated with surface markers during SARS-CoV-2 infection (left) and COVID-19 severity (right). Focusing on NC. Mono, the CD86 and CD123 immunotypes are expanded in convalescent severe individuals, which persisted into late convalescence (boxed in dotted line).

**Supplementary Figure 5.** Loss of CD8^+^ MAIT and CD56^Bright^ NK cells are innate-like responses to SARS-CoV-2 infection but not severity. (A) Frequencies of MAIT immunotypes based on disease stage from early active to late convalescence. CD8^+^ but not CD4^+^ MAIT cells are significantly reduced during SARS-CoV-2 infection, which recover with health. Also, frequencies of different MAIT immunotypes with disease severity. There is little or no significance among severity groups I, II and III. (B) The depletion of CD56^Bright^ NK subpopulation when compared to healthy does not associate with COVID-19 disease severity. Also, CD56^Dim^ NK HLA-DR^+^ immunotype is not strongly correlated to disease severity. Scatter plots depict the means with SEM. ns: not significant, *, p <0.03; **, p <0.002; ***, p <0.0002, ****, p <0.0001 (Kruskal-Wallis test with multiple comparison corrected on each disease severity group versus total healthy).

**Supplementary Figure 6.** Heterogeneous T cell activation during SARS-CoV-2 infection. (A) Frequencies of CD4^+^ and CD8^+^ T cells based on HLA-DR and CD38 cell surface markers. Lymphopenia is apparent during active SARS-CoV-2 infection and T lymphocyte number increases in convalescence. Disease severity grouping further delineates finer structures. The CD8^+^ T cell subpopulations e.g. HLA-DR^+^CD38^+^ CD8^+^ T immunotypes are elevated in COVID-19 severity group IV. Scatter plots depict the means with SEM. ns: not significant, *, p <0.03; **, p <0.002; ***, p <0.0002, ****, p <0.0001 (Kruskal-Wallis test with multiple comparison corrected on each disease severity group versus total healthy). (B) 3-dimensional dotplots of statistically significant HLA-DR^+^CD38^+^ CD8^+^ and HLA-DR^+^CD38^-^ CD4^+^ T immunotype against memory CD45RO^+^ antigen. (C) Gating strategies for memory T cells. (D) Barplot of total memory CD8^+^/CD4^+^ T cells (TCM and TEM) and the CD27 and CD127 surface markers across disease severity groups showing similarities and differences among COVID-19 patients and healthy donors.

**Supplementary Figure 7.** COVID-19 severity is specific to selected Vδ2 T, dendritic cell and non-senescent NKT cells. Overview of frequency changes across the (A) Vδ2 T cells and (B) dendritic cells showing significance to severity based on p-values. Scatter plots depict the means with SEM. ns: not significant,*, p <0.03; **, p <0.002; ***, p <0.0002, ****, p <0.0001 (Kruskal-Wallis test with multiple comparison corrected on each disease severity group versus total healthy). An extreme outlier due to a single patient in cDC1 is encircled. Vδ1 T cells are insignificant and thus not shown. (C) The ratio of cDC2: pDC frequency during COVID-19 infection. (D) Clustering of NKT gated based on CD3^+^CD56^+^Vδ1^-^Vδ2^-^ T cells into invariant NKT (iNKT) and CD1d-unrestricted CD8^+^ NKT-like cells. The three human NKT immunotypes (CD4^+^, CD8^+^ and CD4^-^CD8^-^/DN) express Type I presenting invariant Vα24Jα18 TCR and Type II presenting a diverse TCR repertoire, which we did not define in this work.

**Supplementary Figure 8.** Heterogeneous B cell development during SARS-CoV-2 infection. (A) Scatterplots of B cell frequencies from Naïve to Memory based on COVID-19 severity. Scatterplots depict the means with SEM. ns: not significant, *, p <0.03; **, p <0.002; ***, p <0.0002, ****, p <0.0001 (Kruskal-Wallis test with multiple comparison corrected on each disease severity group versus total healthy). (B) Dotplots of representative B cell immunotypes for either expanded or contracted cell population in response to SARS-CoV-2 infection. The absolute numbers of cells out of 300,000 PBMCs are shown.

Supplementary Figure 9. Timeline of infection and impact on COVID-19 disease severity. The figure is a summary of the timeline of infection versus the severity of COVID-19. The timeline is broken down into four phases (early active, late active, early conv. and late conv.), which were used to bin 327 immune cells. The disease severity is expressed as the rho^2^ value from the correlation of their cell frequencies with the severity score. Shown are only immunotypes which have at least a 3-fold change in frequency in the respective phase (compare figures 7A and 7B) and/or a rho^2^ value > 0.25 for the disease correlation (compare figures 7C and 7D). The key immunotypes with increased blood frequencies are labeled in red, immunotypes with decreased frequency in blue. Immune cell/cytokine interactions are taken from figure 7.

| **Variables** | **Healthy Control (N = 10)** | **COVID-19** | | | | | |
| --- | --- | --- | --- | --- | --- | --- | --- |
|  |  | **Total (N = 77)** | **Statistical testing of total COVID-19 vs healthy controls (two-tailed p-value)** | **MILD (No O_2_ Supplementation) - Groups I and VI (N = 31)** | **MODERATE (O_2_ Supplementation) - Groups II and V (N = 20)** | **SEVERE (O_2_ Supplementation + ICU) - Groups III and IV (N = 26)** | **Statistical testing of severity groups (two-tailed p-value, if applicable)** |
| **Sex - No. (%)** | | | | | | | |
| **Male** | 6 (60.0) | 60 (77.9) | 0.2128 (χ2 test) | 20 (64.5) | 17 (85.0) | 23 (88.5) | 0.0814 (χ2 test) |
| **Female** | 4 (40.0) | 17 (22.1) |  | 11 (35.5) | 3 (15.0) | 3 (11.5) |  |
| **Age - Years** | | | | | | | |
| **Mean ± SD.** | 36.6 ± 9.2 | 50.4 ± 14.7 | 0.007 (MW U test) | 43.0 ± 13.0 | 52.9 ± 13.6 | 57.4 ± 13.9 | <0.0001 (KW test with Dunn's multiple comparison) |
| **Median (IQR)** | 34.0 (30.75-41.25) | 51.0 (39.5-62.0) |  | 44.0 (30.0-54.0) | 53.0 (42.5-62.5) | 60.5 (45.0-66.5) |  |
| **Range** | 25.0-56.0 | 24.0-82.0 |  | 24.0-65.0 | 28.0-80.0 | 29.0-82.0 |  |
| **Time from Onset to Admission, Days** | | | | | | | |
| **Mean ± SD.** |  |  |  | 5.0 ± 8.1 | 5.4 ± 6.6 | 4.8 ± 3.4 | 0.528 (KW test with Dunn's multiple comparison) |
| **Median (IQR)** |  |  |  | 3.0 (1.0-5.0) | 4.0 (2.0-7.0) | 3.0 (2.0-9.0) |  |
| **Range** |  |  |  | 0.0-45.0 | 1.0-31.0 | 0.0-10.0 |  |
| **Time from Admission to ICU, Days** | | | | | | | |
| **Mean ± SD.** |  |  |  |  |  | 2.4 ± 1.8 |  |
| **Median (IQR)** |  |  |  |  |  | 2.0 (1.0-4.0) |  |
| **Range** |  |  |  |  |  | 0.0-6.0 |  |
| **Duration under Intubation, Days** | | | | | | | |
| **Mean ± SD.** |  |  |  |  |  | 7.9 ± 18.8 |  |
| **Median (IQR)** |  |  |  |  |  | 1.5 (0.0-7.75) |  |
| **Range** |  |  |  |  |  | 0.0-95.0* |  |
| **Significant Medical History - No. (%)** | | | | | | | |
| **Myocardial infarction** |  | 12 (15.6) |  | 2 (6.5) | 5 (25.0) | 5 (19.2) | 0.1673 (χ2 test) |
| **Comorbidity - No. (%)†** | | | | | | | |
| **Hypertension** |  | 27 (35.1) |  | 5 (16.1) | 9 (45.0) | 13 (50.0) | 0.8734 (χ2 test) |
| **Hyperlipidemia/ dyslipidemia** |  | 26 (33.8) |  | 3 (9.7) | 11 (55.0) | 12 (46.2) | 0.4603 (χ2 test) |
| **Diabetes mellitus** |  | 18 (23.4) |  | 1 (3.2) | 6 (30.0) | 11 (42.3) | 0.5487 (χ2 test) |

**Supplementary Table 1.** Demographic details of the study subjects. Population variables (sex and age at admission) and clinical variables (time from symptom onset to hospital admission, time from hospital admission to ICU entry, period of intubation, significant medical history and existing comorbidities) are shown. IQR, interquartile range; KW test, Kruskal-Wallis test; MW U test, Mann-Whitney U test; SD, standard deviation. * Includes the sole outlier who was intubated for 95 days before passing away from COVID-19. † Each instance of comorbidity was counted, even in cases where a patient had multiple comorbidities.

|  | **B cells** | **Abbrev.** | **CD45^+^CD3^-^** |
| --- | --- | --- | --- |
| 1 | Naïve | - | CD19^+^CD27^-^IgM^+^IgD^+^ |
| 2 | Transitional | Trans. | CD19^+^CD27^-^IgM^+/bright^CD38^+^CD24^+^ |
| 3 | Non-class-switched memory | NSM | CD19^+^CD27^+^IgM^+^IgD^+^CD38^+/-^ |
| 4 | Class switched memory | CSM | CD19^+^CD27^+^IgM^-^IgD^-^CD38^+/dim^ |
| 5 | IgM memory | IgM Mem | CD19^+^CD27^+^IgM^+^IgD^-^ |
| 6 | Plasmablasts | - | CD19^+^CD27^+/bright^ IgM^-^IgD^-^CD38^+/bright^ or TriMAP cluster |
| 7 | B regulatory | Breg | CD19^+^CD27^-^IgM^+/bright^CD24^+/bright^CD5^+^ |
|  | **T cells** | **Abbrev.** | **CD45^+^CD3^+^** |
| 8,9 | Naïve | - | CD4^+^ or CD8^+^CD45RA^+^CD45RO^-^CCR7^+^CD27^+^ |
| 10,11 | Central memory | CM | CD4^+^ or CD8^+^CD45RA^-^CD45RO^+^CCR7^+^ |
| 12,13 | Effector memory | EM | CD4^+^ or CD8^+^CD45RA^-^CD45RO^+^CCR7^-^ |
| 14,15 | Terminally differentiated effector memory | TEMRA | CD4^+^ or CD8^+^CD45RA^+^CD45RO^-^CCR7^-^CD27^-^ |
| - | T regulatory | Treg | CD4^+^CD25^+^CD127^-/dim^ |
| 16 | Treg naive | - | Treg\| CD45RA^+^CD45RO^-^CCR7^+^CD27^+^ |
| 17 | Treg CM | Treg CM | Treg\| CD45RA^-^CD45RO^+^CCR7^+^ |
| 18 | Treg EM | Treg EM | Treg\| CD45RA^-^CD45RO^+^CCR7^-^ |
| 19 | Treg Terminally differentiated effector memory | Treg TEMRA | Treg\| CD45RA^+^CD45RO^-^CCR7^-^CD27^-^ |
| 20 | Vδ1 T | - | CD4^-^CD8^-^TCR^+^Vδ1^+^ |
| 21 | Vδ2 T | - | CD4^-^CD8^-^TCR^+^Vδ2^+^ or TriMAP cluster |
| 22 | Germinal center resident T follicular helper | T_FH_ | CD4^+^CD25^-^CXCR5^+^CCR7^-^CD127^+^ |
| 23 | Mucosal associated invariant T cells | MAIT | CD14^-^CD19^-^Vα7.2^+^CD161^+/bright^CD56^+/-^ |
| 24 | NKT | NKT | CD14^-^CD19^-^Vα7.2^-^CD56^+^ |
|  | **TriMAP gating** | **Abbrev.** | **From TriMAP clustering** |
| 25 | CD56^Dim^ NK | - | TriMAP\| CD19^-^CD14^-^CD56^+^CD16^+^ |
| 26 | CD56^Bright^ NK | - | TriMAP\| CD19^-^CD14^-^CD56^+^CD16^-^ |
| 27 | CD16^+^ NK | - | TriMAP\| CD19^-^CD14^-^CD56^-^CD16^+^ |
| 28 | Classical Monocytes | C. Mono. | TriMAP\| CD19^-^CD14^+/bright^CD16^-^ |
| 29 | Intermediate Monocytes | Int. Mono. | TriMAP\| CD19^-^CD14^+/bright^CD16^+^ |
| 30 | Non-classical Monocytes | NC. Mono. | TriMAP\| CD19^-^CD14^+/dim^CD16^+/bright^ |
| 31 | Plasmacytoid dendritic cells | pDC | TriMAP\| HLA-DR^+^CD11c^-^CD123^+/bright^CD45RA^+^ |
| 32 | Conventional type 1 dendritic cells | cDC1 | TriMAP\| HLA-DR^+^CD11c^+^CD1c^-^CD141^+^ |
| 33 | Conventional type 2 dendritic cells | cDC2 | TriMAP\| HLA-DR^+^CD11c^+^CD141^-^CD1c^+^ |
| 34 | Low-density neutrophils | LD. Neu | TriMAP\| CD11b^+^CD24^+^ or CD66b^+^CD15^+^CD16^high^CD10^+^CD24^+^ |
|  | **Lineage-negative** | **Lin-** | **CD3^-^CD56^-^CD19^-^HLA-DR^-^CD14^-^CD123^-^CD16^-^** |
| 35 | Basophils | Baso. | Lin^-^CD38^+^CD123^+^ |
| 36 | Innate lymphoid cell type 1 | ILC1 | Lin^-^CD5^+^CD11c^-^(CD4^+^, CD8^+^, CD4^-^CD8^-^) |
| 37 | Innate lymphoid cell type 2 | ILC2 | Lin^-^CD5^-^CD11c^-^CD161^+/bright^ |
| 38 | Innate lymphoid cell type 3 | ILC3 | Lin^-^CD5^-^CD11c^+^ |
|  | **Exclusion** |  |  |
| - | Monocytic dendritic cell 4 | DC4 | TriMAP\| HLA-DR^+^CD11c^+^CD141^-^CD1c^-^ |

**Supplementary Table 2.** Gating strategy used for 38 basic immune cells. For TriMAP gated subpopulations, the distinct clusters are calculated using TripMAP algorithm and confirmed based on their surface markers. (-) DC4 immunotype is excluded in analysis due to extreme low frequency.

| **Index** | **Alphabetical Order** | **Short name** | **Rank_num** | **Rank_name** |
| --- | --- | --- | --- | --- |
| 1 | CyTOF B CELLS | B CELLS | 45 | (45) B cells |
| 2 | CyTOF B CELLS CCR7- | B CELLS CCR7- | 27 | (27) B CCR7- |
| 3 | CyTOF B CELLS CCR7+ | B CELLS CCR7+ | 18 | (18) B CCR7+ |
| 4 | CyTOF B CELLS CD38+ | B CELLS CD38+ | 3 | (3) B CD38+ |
| 5 | CyTOF B CELLS CXCR5- | B CELLS CXCR5- | 8 | (8) B CXCR5- |
| 6 | CyTOF B CELLS CXCR5+ | B CELLS CXCR5+ | 47 | (47) B CXCR5+ |
| 7 | CyTOF B CELLS IgA+CD38++ | B CELLS IgA+CD38high | 7 | (7) B IgA+CD38++ |
| 8 | CyTOF B CELLS IgD-CD27- | B CELLS IgD-CD27- | 16 | (16) B IgD-CD27- |
| 9 | CyTOF B CELLS IgD+ | B CELLS IgD+ | 46 | (46) B IgD+ |
| 10 | CyTOF B CELLS IgD+CD27+ | B CELLS IgD+CD27+ | 12 | (12) B IgD+CD27+ |
| 11 | CyTOF B CELLS IgD+CD38+ | B CELLS IgD+CD38+ | 40 | (40) B IgD+CD38+ |
| 12 | CyTOF B CELLS IgM+ | B CELLS IgM+ | 41 | (41) B IgM+ |
| 13 | CyTOF B CELLS IgM+CD123+ | B CELLS IgM+CD123+ | 28 | (28) B IgM+CD123+ |
| 14 | CyTOF B CELLS IgM+CD38++ | B CELLS IgM+CD38high | 10 | (10) B IgM+CD38++ |
| 15 | CyTOF B CELLS lgA+ | B CELLS lgA+ | 32 | (32) B lgA+ |
| 16 | CyTOF B CELLS MEM CCR7- | B CELLS MEM CCR7- | 24 | (24) B MEM CCR7- |
| 17 | CyTOF B CELLS MEM CCR7+ | B CELLS MEM CCR7+ | 19 | (19) B MEM CCR7+ |
| 18 | CyTOF B CELLS MEM CD38++ | B CELLS MEM CD38high | 2 | (2) B MEM CD38++ |
| 19 | CyTOF B CELLS MEM CXCR5- | B CELLS MEM CXCR5- | 5 | (5) B MEM CXCR5- |
| 20 | CyTOF B CELLS MEM CXCR5+ | B CELLS MEM CXCR5+ | 35 | (35) B MEM CXCR5+ |
| 21 | CyTOF B CELLS MEM IgM+ | B CELLS MEM IgM+ | 36 | (36) B MEM IgM+ |
| 22 | CyTOF B CELLS MEM IgM+CXCR5- | B CELLS MEM IgM+CXCR5- | 1 | (1) B MEM IgM+CXCR5- |
| 23 | CyTOF B CELLS MEM IgM+CXCR5+ | B CELLS MEM IgM+CXCR5+ | 25 | (25) B MEM IgM+CXCR5+ |
| 24 | CyTOF B CELLS NAIVE | B CELLS NAIVE | 42 | (42) B NAIVE |
| 25 | CyTOF B CELLS NAIVE CCR7- | B CELLS NAIVE CCR7- | 37 | (37) B NAIVE CCR7- |
| 26 | CyTOF B CELLS NAIVE CCR7+ | B CELLS NAIVE CCR7+ | 15 | (15) B NAIVE CCR7+ |
| 27 | CyTOF B CELLS NAIVE CD123+ | B CELLS NAIVE CD123+ | 30 | (30) B NAIVE CD123+ |
| 28 | CyTOF B CELLS NAIVE CD5- | B CELLS NAIVE CD5- | 17 | (17) B NAIVE CD5- |
| 29 | CyTOF B CELLS NAIVE CD5+ | B CELLS NAIVE CD5+ | 22 | (22) B NAIVE CD5+ |
| 30 | CyTOF B CELLS NAIVE CXCR5- | B CELLS NAIVE CXCR5- | 20 | (20) B NAIVE CXCR5- |
| 31 | CyTOF B CELLS NAIVE IgM- | B CELLS NAIVE IgM- | 33 | (33) B NAIVE IgM- |
| 32 | CyTOF B CELLS NAIVE IgM+ | B CELLS NAIVE IgM+ | 26 | (26) B NAIVE IgM+ |
| 33 | CyTOF B MEM | B CELLS MEM | 34 | (34) B MEM |
| 34 | CyTOF BASO | BASO | 1 | (1) BASO |
| 35 | CyTOF BREG | BREG | 3 | (3) BREG |
| 36 | CyTOF BREG CD123+ | BREG CD123+ | 1 | (1) BREG CD123+ |
| 37 | CyTOF BREG CD38+ | BREG CD38+ | 2 | (2) BREG CD38+ |
| 38 | CyTOF BREG PDL1 | BREG PDL1 | 4 | (4) BREG PDL1 |
| 39 | CyTOF C. MONO | C. MONO | 17 | (17) C. Mono |
| 40 | CyTOF C. Mono CD141- | C. Mono CD141- | 20 | (20) C. Mono CD141- |
| 41 | CyTOF C. Mono CD141-CD11B- | C. Mono CD141-CD11B- | 15 | (15) C. Mono CD141-CD11B- |
| 42 | CyTOF C. Mono CD141-CD11B+ | C. Mono CD141-CD11B+ | 18 | (18) C. Mono CD141-CD11B+ |
| 43 | CyTOF C. Mono CD141-HLADR- | C. Mono CD141-HLADR- | 7 | (7) C. Mono CD141-HLADR- |
| 44 | CyTOF C. Mono CD141-HLADR+ | C. Mono CD141-HLADR+ | 31 | (31) C. Mono CD141-HLADR+ |
| 45 | CyTOF C. Mono CD141+ | C. Mono CD141+ | 16 | (16) C. Mono CD141+ |
| 46 | CyTOF C. Mono CD141+CD11B- | C. Mono CD141+CD11B- | 13 | (13) C. Mono CD141+CD11B- |
| 47 | CyTOF C. Mono CD141+CD11B+ | C. Mono CD141+CD11B+ | 19 | (19) C. Mono CD141+CD11B+ |
| 48 | CyTOF C. Mono CD141+HLADR- | C. Mono CD141+HLADR- | 4 | (4) C. Mono CD141+HLADR- |
| 49 | CyTOF C. Mono CD141+HLADR+ | C. Mono CD141+HLADR+ | 27 | (27) C. Mono CD141+HLADR+ |
| 50 | CyTOF C. Mono CD16- | C. Mono CD16- | 11 | (11) C. Mono CD16- |
| 51 | CyTOF C. Mono CD16-CD11B- | C. Mono CD16-CD11B- | 22 | (22) C. Mono CD16-CD11B- |
| 52 | CyTOF C. Mono CD16-CD11B+ | C. Mono CD16-CD11B+ | 9 | (9) C. Mono CD16-CD11B+ |
| 53 | CyTOF C. Mono CD16-CD169- | C. Mono CD16-CD169- | 14 | (14) C. Mono CD16-CD169- |
| 54 | CyTOF C. Mono CD16-CD169+ | C. Mono CD16-CD169+ | 6 | (6) C. Mono CD16-CD169+ |
| 55 | CyTOF C. Mono CD16+ | C. Mono CD16+ | 24 | (24) C. Mono CD16+ |
| 56 | CyTOF C. Mono CD16+CD11B- | C. Mono CD16+CD11B- | 10 | (10) C. Mono CD16+CD11B- |
| 57 | CyTOF C. Mono CD16+CD11B+ | C. Mono CD16+CD11B+ | 26 | (26) C. Mono CD16+CD11B+ |
| 58 | CyTOF C. Mono CD16+CD169- | C. Mono CD16+CD169- | 23 | (23) C. Mono CD16+CD169- |
| 59 | CyTOF C. Mono CD16+CD169+ | C. Mono CD16+CD169+ | 25 | (25) C. Mono CD16+CD169+ |
| 60 | CyTOF C. Mono CD169-HLADR- | C. Mono CD169-HLADR- | 5 | (5) C. Mono CD169-HLADR- |
| 61 | CyTOF C. Mono CD169-HLADR+ | C. Mono CD169-HLADR+ | 28 | (28) C. Mono CD169-HLADR+ |
| 62 | CyTOF C. MONO CD169+ | C. MONO CD169+ | 3 | (3) C. Mono CD169+ |
| 63 | CyTOF C. Mono CD169+HLADR- | C. Mono CD169+HLADR- | 1 | (1) C. Mono CD169+HLADR- |
| 64 | CyTOF C. Mono CD169+HLADR+ | C. Mono CD169+HLADR+ | 29 | (29) C. Mono CD169+HLADR+ |
| 65 | CyTOF C. MONO CD38+ | C. MONO CD38+ | 21 | (21) C. Mono CD38+ |
| 66 | CyTOF C. MONO CD45RA- | C. MONO CD45RA- | 12 | (12) C. Mono CD45RA- |
| 67 | CyTOF C. MONO CD45RA+ | C. MONO CD45RA+ | 30 | (30) C. Mono CD45RA+ |
| 68 | CyTOF C. MONO CD56+ | C. MONO CD56+ | 8 | (8) C. Mono CD56+ |
| 69 | CyTOF C. MONO CD86+ | C. MONO CD86+ | 32 | (32) C. Mono CD86+ |
| 70 | CyTOF C. MONO IgA+ | C. MONO IgA+ | 2 | (2) C. Mono IgA+ |
| 71 | CyTOF CD141+HLADR-Cells | CD141+HLADR-Cells | 1 | (1) CD141+HLADR- Cells |
| 72 | CyTOF CD16+ NK | CD16+ NK | 1 | (1) CD16+ NK |
| 73 | CyTOF CD16+ NK CD38+ | CD16+ NK CD38+ | 2 | (2) CD16+ NK CD38+ |
| 74 | CyTOF CD4 CD11C+ | CD4 CD11C+ | 10 | (10) CD4 CD11C+ |
| 75 | CyTOF CD4 CD127+ | CD4 CD127+ | 18 | (18) CD4 CD127+ |
| 76 | CyTOF CD4 CD38+ | CD4 CD38+ | 24 | (24) CD4 CD38+ |
| 77 | CyTOF CD4 CD57+ | CD4 CD57+ | 33 | (33) CD4 CD57+ |
| 78 | CyTOF CD4 CXCR5- | CD4 CXCR5- | 20 | (20) CD4 CXCR5- |
| 79 | CyTOF CD4 CXCR5+ | CD4 CXCR5+ | 13 | (13) CD4 CXCR5+ |
| 80 | CyTOF CD4 HLADR- CD38+CD45RO+ | CD4 HLADR- CD38+CD45RO+ | 34 | (34) CD4 HLADR- CD38+CD45RO+ |
| 81 | CyTOF CD4 HLADR-CD38- | CD4 HLADR-CD38- | 12 | (12) CD4 HLADR-CD38- |
| 82 | CyTOF CD4 HLADR-CD38+ | CD4 HLADR-CD38+ | 21 | (21) CD4 HLADR-CD38+ |
| 83 | CyTOF CD4 HLADR+ CD38+CD45RO+ | CD4 HLADR+ CD38+CD45RO+ | 7 | (7) CD4 HLADR+ CD38+CD45RO+ |
| 84 | CyTOF CD4 HLADR+CD38- | CD4 HLADR+CD38- | 1 | (1) CD4 HLADR+CD38- |
| 85 | CyTOF CD4 HLADR+CD38+ | CD4 HLADR+CD38+ | 30 | (30) CD4 HLADR+CD38+ |
| 86 | CyTOF CD4 NAIVE | CD4 NAIVE | 15 | (15) CD4 NAIVE |
| 87 | CyTOF CD4 NAIVE CD127+ | CD4 NAIVE CD127+ | 16 | (16) CD4 NAIVE CD127+ |
| 88 | CyTOF CD4 NAIVE CD27+ | CD4 NAIVE CD27+ | 14 | (14) CD4 NAIVE CD27+ |
| 89 | CyTOF CD4 NAIVE CD38+ | CD4 NAIVE CD38+ | 4 | (4) CD4 NAIVE CD38+ |
| 90 | CyTOF CD4 T | CD4 T | 19 | (19) CD4 T |
| 91 | CyTOF CD4 TCM | CD4 TCM | 27 | (27) CD4 TCM |
| 92 | CyTOF CD4 TCM CD127+ | CD4 TCM CD127+ | 22 | (22) CD4 TCM CD127+ |
| 93 | CyTOF CD4 TCM CD27+ | CD4 TCM CD27+ | 25 | (25) CD4 TCM CD27+ |
| 94 | CyTOF CD4 TCM CD38+ | CD4 TCM CD38+ | 28 | (28) CD4 TCM CD38+ |
| 95 | CyTOF CD4 TCM CD38+HLADR+ | CD4 TCM CD38+HLADR+ | 3 | (3) CD4 TCM CD38+HLADR+ |
| 96 | CyTOF CD4 TEM | CD4 TEM | 29 | (29) CD4 TEM |
| 97 | CyTOF CD4 TEM CD127+ | CD4 TEM CD127+ | 26 | (26) CD4 TEM CD127+ |
| 98 | CyTOF CD4 TEM CD38+ | CD4 TEM CD38+ | 8 | (8) CD4 TEM CD38+ |
| 99 | CyTOF CD4 TEM CD38+HLADR+ | CD4 TEM CD38+HLADR+ | 5 | (5) CD4 TEM CD38+HLADR+ |
| 100 | CyTOF CD4 TEMRA | CD4 TEMRA | 17 | (17) CD4 TEMRA |
| 101 | CyTOF CD4 TEMRA CD27- | CD4 TEMRA CD27- | 2 | (2) CD4 TEMRA CD27- |
| 102 | CyTOF CD4 TEMRA CD38+ | CD4 TEMRA CD38+ | 31 | (31) CD4 TEMRA CD38+ |
| 103 | CyTOF CD4+ CD56- MAIT | CD4+ CD56- MAIT | 6 | (6) CD4+ CD56- MAIT |
| 104 | CyTOF CD4+ CD56- MAIT CD38+ | CD4+ CD56- MAIT CD38+ | 9 | (9) CD4+ CD56- MAIT CD38+ |
| 105 | CyTOF CD4+ CD56+ MAIT | CD4+ CD56+ MAIT | 23 | (23) CD4+ CD56+ MAIT |
| 106 | CyTOF CD4+ CD56+ MAIT CD38+ | CD4+ CD56+ MAIT CD38+ | 32 | (32) CD4+ CD56+ MAIT CD38+ |
| 107 | CyTOF CD4+CD25+ | CD4+CD25+ | 11 | (11) CD4+CD25+ |
| 108 | CyTOF CD56 BRIGHT NK | CD56 BRIGHT NK | 1 | (1) CD56 BRIGHT NK |
| 109 | CyTOF CD56 BRIGHT NK CD11B+ | CD56 BRIGHT NK CD11B+ | 4 | (4) CD56 BRIGHT NK CD11B+ |
| 110 | CyTOF CD56 BRIGHT NK CD11C+ | CD56 BRIGHT NK CD11C+ | 6 | (6) CD56 BRIGHT NK CD11C+ |
| 111 | CyTOF CD56 BRIGHT NK CD38+ | CD56 BRIGHT NK CD38+ | 5 | (5) CD56 BRIGHT NK CD38+ |
| 112 | CyTOF CD56 BRIGHT NK CD5+ | CD56 BRIGHT NK CD5+ | 2 | (2) CD56 BRIGHT NK CD5+ |
| 113 | CyTOF CD56 BRIGHT NK HLADR+ | CD56 BRIGHT NK HLADR+ | 3 | (3) CD56 BRIGHT NK HLADR+ |
| 114 | CyTOF CD56DIM NK | CD56DIM NK | 1 | (1) CD56DIM NK |
| 115 | CyTOF CD56DIM NK CD11B+ | CD56DIM NK CD11B+ | 2 | (2) CD56DIM NK CD11B+ |
| 116 | CyTOF CD56DIM NK CD11C+ | CD56DIM NK CD11C+ | 3 | (3) CD56DIM NK CD11C+ |
| 117 | CyTOF CD56DIM NK CD38+ | CD56DIM NK CD38+ | 4 | (4) CD56DIM NK CD38+ |
| 118 | CyTOF CD56DIM NK CD5+ | CD56DIM NK CD5+ | 5 | (5) CD56DIM NK CD5+ |
| 119 | CyTOF CD56DIM NK HLADR+ | CD56DIM NK HLADR+ | 6 | (6) CD56DIM NK HLADR+ |
| 120 | CyTOF CD8 CD11C+ | CD8 CD11C+ | 12 | (12) CD8 CD11C+ |
| 121 | CyTOF CD8 CD38+ | CD8 CD38+ | 23 | (23) CD8 CD38+ |
| 122 | CyTOF CD8 CXCR5- | CD8 CXCR5- | 16 | (16) CD8 CXCR5- |
| 123 | CyTOF CD8 CXCR5+ | CD8 CXCR5+ | 10 | (10) CD8 CXCR5+ |
| 124 | CyTOF CD8 HLADR- CD38+CD45RO+ | CD8 HLADR- CD38+CD45RO+ | 24 | (24) CD8 HLADR- CD38+CD45RO+ |
| 125 | CyTOF CD8 HLADR-CD38- | CD8 HLADR-CD38- | 6 | (6) CD8 HLADR-CD38- |
| 126 | CyTOF CD8 HLADR-CD38+ | CD8 HLADR-CD38+ | 21 | (21) CD8 HLADR-CD38+ |
| 127 | CyTOF CD8 HLADR+ CD38+CD45RO+ | CD8 HLADR+ CD38+CD45RO+ | 26 | (26) CD8 HLADR+ CD38+CD45RO+ |
| 128 | CyTOF CD8 HLADR+CD38- | CD8 HLADR+CD38- | 1 | (1) CD8 HLADR+CD38- |
| 129 | CyTOF CD8 HLADR+CD38+ | CD8 HLADR+CD38+ | 28 | (28) CD8 HLADR+CD38+ |
| 130 | CyTOF CD8 NAIVE | CD8 NAIVE | 15 | (15) CD8 NAIVE |
| 131 | CyTOF CD8 NAIVE CD127+ | CD8 NAIVE CD127+ | 19 | (19) CD8 NAIVE CD127+ |
| 132 | CyTOF CD8 NAIVE CD27+ | CD8 NAIVE CD27+ | 17 | (17) CD8 NAIVE CD27+ |
| 133 | CyTOF CD8 NAIVE CD38+ | CD8 NAIVE CD38+ | 7 | (7) CD8 NAIVE CD38+ |
| 134 | CyTOF CD8 T CELL | CD8 T CELL | 13 | (13) CD8 T CELL |
| 135 | CyTOF CD8 TCM CD127+ | CD8 TCM CD127+ | 5 | (5) CD8 TCM CD127+ |
| 136 | CyTOF CD8 TCM CD27+ | CD8 TCM CD27+ | 14 | (14) CD8 TCM CD27+ |
| 137 | CyTOF CD8 TCM CD38+ | CD8 TCM CD38+ | 29 | (29) CD8 TCM CD38+ |
| 138 | CyTOF CD8 TCM CD38+HLADR+ | CD8 TCM CD38+HLADR+ | 27 | (27) CD8 TCM CD38+HLADR+ |
| 139 | CyTOF CD8 TEM | CD8 TEM | 20 | (20) CD8 TEM |
| 140 | CyTOF CD8 TEM CD127+ | CD8 TEM CD127+ | 3 | (3) CD8 TEM CD127+ |
| 141 | CyTOF CD8 TEM CD27+ | CD8 TEM CD27+ | 22 | (22) CD8 TEM CD27+ |
| 142 | CyTOF CD8 TEM CD38+ | CD8 TEM CD38+ | 30 | (30) CD8 TEM CD38+ |
| 143 | CyTOF CD8 TEM CD38+HLADR+ | CD8 TEM CD38+HLADR+ | 25 | (25) CD8 TEM CD38+HLADR+ |
| 144 | CyTOF CD8 TEMRA | CD8 TEMRA | 8 | (8) CD8 TEMRA |
| 145 | CyTOF CD8 TEMRA CD127+ | CD8 TEMRA CD127+ | 2 | (2) CD8 TEMRA CD127+ |
| 146 | CyTOF CD8 TEMRA CD27- | CD8 TEMRA CD27- | 4 | (4) CD8 TEMRA CD27- |
| 147 | CyTOF CD8 TEMRA CD38+ | CD8 TEMRA CD38+ | 11 | (11) CD8 TEMRA CD38+ |
| 148 | CyTOF CD8+ CD25+ | CD8+ CD25+ | 9 | (9) CD8+ CD25+ |
| 149 | CyTOF CD8+ CD56- MAIT | CD8+ CD56- MAIT | 2 | (2) MAIT CD8+ CD56- |
| 150 | CyTOF CD8+ CD56- MAIT CD38+ | CD8+ CD56- MAIT CD38+ | 1 | (1) MAIT CD8+ CD56- CD38+ |
| 151 | CyTOF CD8+ CD56+ MAIT | CD8+ CD56+ MAIT | 5 | (5) MAIT CD8+ CD56+ |
| 152 | CyTOF CD8+ CD56+ MAIT CD38+ | CD8+ CD56+ MAIT CD38+ | 10 | (10) MAIT CD8+ CD56+ CD38+ |
| 153 | CyTOF CD8+ TCM | CD8+ TCM | 18 | (18) CD8+ TCM |
| 154 | CyTOF cDC1 | cDC1 | 7 | (7) cDC1 |
| 155 | CyTOF cDC1 CD38+ | cDC1 CD38+ | 8 | (8) cDC1 CD38+ |
| 156 | CyTOF cDC2 | cDC2 | 2 | (2) cDC2 |
| 157 | CyTOF cDC2 CD11B+ | cDC2 CD11B+ | 6 | (6) cDC2 CD11B+ |
| 158 | CyTOF cDC2 CD38+ | cDC2 CD38+ | 3 | (3) cDC2 CD38+ |
| 159 | CyTOF cDC2 CD5- | cDC2 CD5- | 4 | (4) cDC2 CD5- |
| 160 | CyTOF cDC2 CD5+ | cDC2 CD5+ | 5 | (5) cDC2 CD5+ |
| 161 | CyTOF CSM | CSM | 43 | (43) B CSM |
| 162 | CyTOF CSM CCR6- | CSM CCR6- | 29 | (29) B CSM CCR6- |
| 163 | CyTOF CSM CCR6+ | CSM CCR6+ | 39 | (39) B CSM CCR6+ |
| 164 | CyTOF CSM CXCR5- | CSM CXCR5- | 31 | (31) B CSM CXCR5- |
| 165 | CyTOF CSM CXCR5+ | CSM CXCR5+ | 38 | (38) B CSM CXCR5+ |
| 166 | CyTOF DC | DC | 1 | (1) DC |
| 167 | CyTOF DC4 | DC4 | 9 | (9) cDC4 |
| 168 | CyTOF DC4 CD38+ | DC4 CD38+ | 10 | (10) cDC4 CD38+ |
| 169 | CyTOF DN CD56- MAIT | DN CD56- MAIT | 3 | (3) MAIT DN CD56- |
| 170 | CyTOF DN CD56- MAIT CD38+ | DN CD56- MAIT CD38+ | 6 | (6) MAIT DN CD56- CD38+ |
| 171 | CyTOF DN CD56+ MAIT | DN CD56+ MAIT | 4 | (4) MAIT DN CD56+ |
| 172 | CyTOF DN CD56+ MAIT CD38+ | DN CD56+ MAIT CD38+ | 11 | (11) MAIT DN CD56+ CD38+ |
| 173 | CyTOF ICOS+ TFH | ICOS+ TFH | 3 | (3) TFH ICOS+ |
| 174 | CyTOF ILC1 | ILC1 | 5 | (5) ILC1 |
| 175 | CyTOF ILC1 CD25- | ILC1 CD25- | 6 | (6) ILC1 CD25- |
| 176 | CyTOF ILC1 CD25+ | ILC1 CD25+ | 7 | (7) ILC1 CD25+ |
| 177 | CyTOF ILC1 CD38+ | ILC1 CD38+ | 8 | (8) ILC1 CD38+ |
| 178 | CyTOF ILC2 | ILC2 | 1 | (1) ILC2 |
| 179 | CyTOF ILC2 CD25- | ILC2 CD25- | 2 | (2) ILC2 CD25- |
| 180 | CyTOF ILC2 CD25+ | ILC2 CD25+ | 3 | (3) ILC2 CD25+ |
| 181 | CyTOF ILC2 CD38+ | ILC2 CD38+ | 4 | (4) ILC2 CD38+ |
| 182 | CyTOF ILC3 | ILC3 | 9 | (9) ILC3 |
| 183 | CyTOF ILC3 CD25- | ILC3 CD25- | 10 | (10) ILC3 CD25- |
| 184 | CyTOF ILC3 CD25+ | ILC3 CD25+ | 11 | (11) ILC3 CD25+ |
| 185 | CyTOF INT. MONO | INT. MONO | 14 | (14) INT. Mono |
| 186 | CyTOF Int. Mono CD141- | Int. Mono CD141- | 24 | (24) INT. Mono CD141- |
| 187 | CyTOF Int. Mono CD141-CD11B- | Int. Mono CD141-CD11B- | 18 | (18) INT. Mono CD141-CD11B- |
| 188 | CyTOF Int. Mono CD141-CD11B+ | Int. Mono CD141-CD11B+ | 17 | (17) INT. Mono CD141-CD11B+ |
| 189 | CyTOF Int. Mono CD141-HLADR- | Int. Mono CD141-HLADR- | 7 | (7) INT. Mono CD141-HLADR- |
| 190 | CyTOF Int. Mono CD141-HLADR+ | Int. Mono CD141-HLADR+ | 21 | (21) INT. Mono CD141-HLADR+ |
| 191 | CyTOF Int. Mono CD141+ | Int. Mono CD141+ | 11 | (11) INT. Mono CD141+ |
| 192 | CyTOF Int. Mono CD141+CD11B- | Int. Mono CD141+CD11B- | 10 | (10) INT. Mono CD141+CD11B- |
| 193 | CyTOF Int. Mono CD141+CD11B+ | Int. Mono CD141+CD11B+ | 16 | (16) INT. Mono CD141+CD11B+ |
| 194 | CyTOF Int. Mono CD141+HLADR- | Int. Mono CD141+HLADR- | 2 | (2) INT. Mono CD141+HLADR- |
| 195 | CyTOF Int. Mono CD141+HLADR+ | Int. Mono CD141+HLADR+ | 19 | (19) INT. Mono CD141+HLADR+ |
| 196 | CyTOF Int. Mono CD16+ | Int. Mono CD16+ | 8 | (8) INT. Mono CD16+ |
| 197 | CyTOF Int. Mono CD16++ | Int. Mono CD16++ | 22 | (22) INT. Mono CD16++ |
| 198 | CyTOF Int. Mono CD16+CD11B- | Int. Mono CD16+CD11B- | 13 | (13) INT. Mono CD16+CD11B- |
| 199 | CyTOF Int. Mono CD16+CD11B+ | Int. Mono CD16+CD11B+ | 15 | (15) INT. Mono CD16+CD11B+ |
| 200 | CyTOF Int. Mono CD16+CD169- | Int. Mono CD16+CD169- | 9 | (9) INT. Mono CD16+CD169- |
| 201 | CyTOF Int. Mono CD16+CD169+ | Int. Mono CD16+CD169+ | 25 | (25) INT. Mono CD16+CD169+ |
| 202 | CyTOF Int. Mono CD169-HLADR- | Int. Mono CD169-HLADR- | 4 | (4) INT. Mono CD169-HLADR- |
| 203 | CyTOF Int. Mono CD169-HLADR+ | Int. Mono CD169-HLADR+ | 12 | (12) INT. Mono CD169-HLADR+ |
| 204 | CyTOF INT. MONO CD169+ | INT. MONO CD169+ | 23 | (23) INT. Mono CD169+ |
| 205 | CyTOF Int. Mono CD169+HLADR- | Int. Mono CD169+HLADR- | 1 | (1) INT. Mono CD169+HLADR- |
| 206 | CyTOF Int. Mono CD169+HLADR+ | Int. Mono CD169+HLADR+ | 26 | (26) INT. Mono CD169+HLADR+ |
| 207 | CyTOF INT. MONO CD38+ | INT. MONO CD38+ | 20 | (20) INT. Mono CD38+ |
| 208 | CyTOF INT. MONO CD45RA- | INT. MONO CD45RA- | 6 | (6) INT. Mono CD45RA- |
| 209 | CyTOF INT. MONO CD45RA+ | INT. MONO CD45RA+ | 28 | (28) INT. Mono CD45RA+ |
| 210 | CyTOF INT. MONO CD56+ | INT. MONO CD56+ | 5 | (5) INT. Mono CD56+ |
| 211 | CyTOF INT. MONO CD86+ | INT. MONO CD86+ | 27 | (27) INT. Mono CD86+ |
| 212 | CyTOF INT. MONO IgA+ | INT. MONO IgA+ | 3 | (3) INT. Mono IgA+ |
| 213 | CyTOF LD Neu | LD Neu | 1 | (1) LD Neu |
| 214 | CyTOF LD Neu CD38+ | LD Neu CD38+ | 2 | (2) LD Neu CD38+ |
| 215 | CyTOF LD Neu CD5+ | LD Neu CD5+ | 3 | (3) LD Neu CD5+ |
| 216 | CyTOF LD Neu IgA+ | LD Neu IgA+ | 4 | (4) LD Neu IgA+ |
| 217 | CyTOF LD Neu PD-L1+ | LD Neu PD-L1+ | 5 | (5) LD Neu PD-L1+ |
| 218 | CyTOF MAIT | MAIT | 8 | (8) MAIT |
| 219 | CyTOF MAIT CD38+ | MAIT CD38+ | 13 | (13) MAIT CD38+ |
| 220 | CyTOF MAIT CD56- | MAIT CD56- | 9 | (9) MAIT CD56- |
| 221 | CyTOF MAIT CD56- CD38+ | MAIT CD56- CD38+ | 14 | (14) MAIT CD56- CD38+ |
| 222 | CyTOF MAIT CD56+ | MAIT CD56+ | 7 | (7) MAIT CD56+ |
| 223 | CyTOF MAIT CD56+ CD38+ | MAIT CD56+ CD38+ | 12 | (12) MAIT CD56+ CD38+ |
| 224 | CyTOF NC. MONO | NC. MONO | 16 | (16) NC. Mono |
| 225 | CyTOF NC. MONO CCR7- | NC. MONO CCR7- | 17 | (17) NC. Mono CCR7- |
| 226 | CyTOF NC. MONO CCR7+ | NC. MONO CCR7+ | 6 | (6) NC. Mono CCR7+ |
| 227 | CyTOF NC. MONO CD123+ | NC. MONO CD123+ | 13 | (13) NC. Mono CD123+ |
| 228 | CyTOF NC. Mono CD141- | NC. Mono CD141- | 20 | (20) NC. Mono CD141- |
| 229 | CyTOF NC. Mono CD141-CD11B- | NC. Mono CD141-CD11B- | 8 | (8) NC. Mono CD141-CD11B- |
| 230 | CyTOF NC. Mono CD141-CD11B+ | NC. Mono CD141-CD11B+ | 24 | (24) NC. Mono CD141-CD11B+ |
| 231 | CyTOF NC. Mono CD141-HLADR- | NC. Mono CD141-HLADR- | 2 | (2) NC. Mono CD141-HLADR- |
| 232 | CyTOF NC. Mono CD141-HLADR+ | NC. Mono CD141-HLADR+ | 21 | (21) NC. Mono CD141-HLADR+ |
| 233 | CyTOF NC. Mono CD141+ | NC. Mono CD141+ | 14 | (14) NC. Mono CD141+ |
| 234 | CyTOF NC. Mono CD141+CD11B- | NC. Mono CD141+CD11B- | 1 | (1) NC. Mono CD141+CD11B- |
| 235 | CyTOF NC. Mono CD141+CD11B+ | NC. Mono CD141+CD11B+ | 23 | (23) NC. Mono CD141+CD11B+ |
| 236 | CyTOF NC. Mono CD141+HLADR- | NC. Mono CD141+HLADR- | 7 | (7) NC. Mono CD141+HLADR- |
| 237 | CyTOF NC. Mono CD141+HLADR+ | NC. Mono CD141+HLADR+ | 15 | (15) NC. Mono CD141+HLADR+ |
| 238 | CyTOF NC. Mono CD16++ | NC. Mono CD16++ | 19 | (19) NC. Mono CD16++ |
| 239 | CyTOF NC. Mono CD16+CD11B- | NC. Mono CD16+CD11B- | 4 | (4) NC. Mono CD16+CD11B- |
| 240 | CyTOF NC. Mono CD16+CD11B+ | NC. Mono CD16+CD11B+ | 25 | (25) NC. Mono CD16+CD11B+ |
| 241 | CyTOF NC. Mono CD16+CD169- | NC. Mono CD16+CD169- | 12 | (12) NC. Mono CD16+CD169- |
| 242 | CyTOF NC. Mono CD16+CD169+ | NC. Mono CD16+CD169+ | 29 | (29) NC. Mono CD16+CD169+ |
| 243 | CyTOF NC. Mono CD169-HLADR- | NC. Mono CD169-HLADR- | 5 | (5) NC. Mono CD169-HLADR- |
| 244 | CyTOF NC. Mono CD169-HLADR+ | NC. Mono CD169-HLADR+ | 11 | (11) NC. Mono CD169-HLADR+ |
| 245 | CyTOF NC. Mono CD169+HLADR- | NC. Mono CD169+HLADR- | 18 | (18) NC. Mono CD169+HLADR- |
| 246 | CyTOF NC. Mono CD169+HLADR+ | NC. Mono CD169+HLADR+ | 27 | (27) NC. Mono CD169+HLADR+ |
| 247 | CyTOF NC. Mono CD16lo | NC. Mono CD16lo | 9 | (9) NC. Mono CD16lo |
| 248 | CyTOF NC. MONO CD38+ | NC. MONO CD38+ | 26 | (26) NC. Mono CD38+ |
| 249 | CyTOF NC. MONO CD45RO- | NC. MONO CD45RO- | 3 | (3) NC. Mono CD45RO- |
| 250 | CyTOF NC. MONO CD45RO+ | NC. MONO CD45RO+ | 30 | (30) NC. Mono CD45RO+ |
| 251 | CyTOF NC. MONO CD56+ | NC. MONO CD56+ | 22 | (22) NC. Mono CD56+ |
| 252 | CyTOF NC. MONO CD86+ | NC. MONO CD86+ | 10 | (10) NC. Mono CD86+ |
| 253 | CyTOF NC. MONO IgA+ | NC. MONO IgA+ | 28 | (28) NC. Mono IgA+ |
| 254 | CyTOF NK CD11B+ | NK CD11B+ | 1 | (1) NK CD11B+ |
| 255 | CyTOF NK CD11C+ | NK CD11C+ | 2 | (2) NK CD11C+ |
| 256 | CyTOF NKT | NKT | 1 | (1) NKT |
| 257 | CyTOF NKT CD161- | NKT CD161- | 2 | (2) NKT CD161- |
| 258 | CyTOF NKT CD161+ | NKT CD161+ | 3 | (3) NKT CD161+ |
| 259 | CyTOF NKT CD38+ | NKT CD38+ | 4 | (4) NKT CD38+ |
| 260 | CyTOF NKT CD57- | NKT CD57- | 5 | (5) NKT CD57- |
| 261 | CyTOF NKT CD57-CD4+ | NKT CD57-CD4+ | 6 | (6) NKT CD57-CD4+ |
| 262 | CyTOF NKT CD57-CD8+ | NKT CD57-CD8+ | 7 | (7) NKT CD57-CD8+ |
| 263 | CyTOF NKT CD57-DN | NKT CD57-DN | 8 | (8) NKT CD57-DN |
| 264 | CyTOF NKT CD57-DP | NKT CD57-DP | 9 | (9) NKT CD57-DP |
| 265 | CyTOF NKT CD57+ | NKT CD57+ | 10 | (10) NKT CD57+ |
| 266 | CyTOF NKT CD57+CD4+ | NKT CD57+CD4+ | 11 | (11) NKT CD57+CD4+ |
| 267 | CyTOF NKT CD57+CD8+ | NKT CD57+CD8+ | 12 | (12) NKT CD57+CD8+ |
| 268 | CyTOF NKT CD57+DN | NKT CD57+DN | 13 | (13) NKT CD57+DN |
| 269 | CyTOF NKT CD57+DP | NKT CD57+DP | 14 | (14) NKT CD57+DP |
| 270 | CyTOF NSM | NSM | 14 | (14) B NSM |
| 271 | CyTOF NSM CD27+CD38- | NSM CD27+CD38- | 6 | (6) B NSM CD27+CD38- |
| 272 | CyTOF NSM CD27+CD38+ | NSM CD27+CD38+ | 13 | (13) B NSM CD27+CD38+ |
| 273 | CyTOF NSM CD38+ | NSM CD38+ | 23 | (23) B NSM CD38+ |
| 274 | CyTOF PDC | PDC | 1 | (1) PDC |
| 275 | CyTOF PDC CD45RA+ | PDC CD45RA+ | 2 | (2) PDC CD45RA+ |
| 276 | CyTOF PDC CD45RA+CD38++ | PDC CD45RA+CD38high | 3 | (3) PDC CD45RA+CD38++ |
| 277 | CyTOF PLASMABLASTS | PLASMABLASTS | 3 | (3) PLASMABL. |
| 278 | CyTOF PLASMABLASTS CXCR5- | PLASMABLASTS CXCR5- | 2 | (2) PLASMABL. CXCR5- |
| 279 | CyTOF PLASMABLASTS CXCR5+ | PLASMABLASTS CXCR5+ | 5 | (5) PLASMABL. CXCR5+ |
| 280 | CyTOF PLASMABLASTS IgA- | PLASMABLASTS IgA- | 1 | (1) PLASMABL. IgA- |
| 281 | CyTOF PLASMABLASTS IgA+ | PLASMABLASTS IgA+ | 4 | (4) PLASMABL. IgA+ |
| 282 | CyTOF T CD38+ | T CD38+ | 6 | (6) T CD38+ |
| 283 | CyTOF T cells | T cells | 3 | (3) T cells |
| 284 | CyTOF T cells CCR7- | T cells CCR7- | 1 | (1) T CCR7- |
| 285 | CyTOF T cells CCR7+ | T cells CCR7+ | 5 | (5) T CCR7+ |
| 286 | CyTOF T CXCR5- | T CXCR5- | 4 | (4) T CXCR5- |
| 287 | CyTOF T CXCR5+ | T CXCR5+ | 2 | (2) T CXCR5+ |
| 288 | CyTOF TFH | TFH | 1 | (1) TFH |
| 289 | CyTOF TFH CD38+ | TFH CD38+ | 2 | (2) TFH CD38+ |
| 290 | CyTOF TRANS | TRANS | 11 | (11) B TRANS |
| 291 | CyTOF TRANS CCR6- | TRANS CCR6- | 44 | (44) B TRANS CCR6- |
| 292 | CyTOF TRANS CCR6+ | TRANS CCR6+ | 9 | (9) B TRANS CCR6+ |
| 293 | CyTOF TRANS CD5- | TRANS CD5- | 4 | (4) B TRANS CD5- |
| 294 | CyTOF TRANS CD5+ | TRANS CD5+ | 21 | (21) B TRANS CD5+ |
| 295 | CyTOF TREG | TREG | 3 | (3) TREG |
| 296 | CyTOF TREG CD11C+ | TREG CD11C+ | 1 | (1) TREG CD11C+ |
| 297 | CyTOF TREG CD38+ | TREG CD38+ | 11 | (11) TREG CD38+ |
| 298 | CyTOF TREG CD45RO-ICOS+CD127+ | TREG CD45RO-ICOS+CD127+ | 6 | (6) TREG CD45RO-ICOS+CD127+ |
| 299 | CyTOF TREG CD45RO+ICOS+CD127+ | TREG CD45RO+ICOS+CD127+ | 9 | (9) TREG CD45RO+ICOS+CD127+ |
| 300 | CyTOF TREG ICOS+ | TREG ICOS+ | 7 | (7) TREG ICOS+ |
| 301 | CyTOF TREG NAIVE | TREG NAIVE | 4 | (4) TREG NAIVE |
| 302 | CyTOF TREG NAIVE CD38+ | TREG NAIVE CD38+ | 5 | (5) TREG NAIVE CD38+ |
| 303 | CyTOF TREG TCM | TREG TCM | 2 | (2) TREG TCM |
| 304 | CyTOF TREG TCM CD38+ | TREG TCM CD38+ | 10 | (10) TREG TCM CD38+ |
| 305 | CyTOF TREG TEM | TREG TEM | 8 | (8) TREG TEM |
| 306 | CyTOF TREG TEM CD38+ | TREG TEM CD38+ | 13 | (13) TREG TEM CD38+ |
| 307 | CyTOF TREG TEMRA | TREG TEMRA | 12 | (12) TREG TEMRA |
| 308 | CyTOF VD1 | VD1 | 11 | (11) VD1 |
| 309 | CyTOF VD1 CD38+ | VD1 CD38+ | 12 | (12) VD1 CD38+ |
| 310 | CyTOF VD1 NAIVE | VD1 NAIVE | 13 | (13) VD1 NAIVE |
| 311 | CyTOF VD1 NAIVE CD38+ | VD1 NAIVE CD38+ | 14 | (14) VD1 NAIVE CD38+ |
| 312 | CyTOF VD1 TCM | VD1 TCM | 15 | (15) VD1 TCM |
| 313 | CyTOF VD1 TCM CD38+ | VD1 TCM CD38+ | 16 | (16) VD1 TCM CD38+ |
| 314 | CyTOF VD1 TEM | VD1 TEM | 17 | (17) VD1 TEM |
| 315 | CyTOF VD1 TEM CD38+ | VD1 TEM CD38+ | 18 | (18) VD1 TEM CD38+ |
| 316 | CyTOF VD1 TEMRA | VD1 TEMRA | 19 | (19) VD1 TEMRA |
| 317 | CyTOF VD1 TEMRA CD38+ | VD1 TEMRA CD38+ | 20 | (20) VD1 TEMRA CD38+ |
| 318 | CyTOF VD2 | VD2 | 1 | (1) VD2 |
| 319 | CyTOF VD2 CD38++ | VD2 CD38high | 2 | (2) VD2 CD38++ |
| 320 | CyTOF VD2 NAIVE | VD2 NAIVE | 3 | (3) VD2 NAIVE |
| 321 | CyTOF VD2 NAIVE CD38+ | VD2 NAIVE CD38+ | 4 | (4) VD2 NAIVE CD38+ |
| 322 | CyTOF VD2 TCM | VD2 TCM | 5 | (5) VD2 TCM |
| 323 | CyTOF VD2 TCM CD38+ | VD2 TCM CD38+ | 6 | (6) VD2 TCM CD38+ |
| 324 | CyTOF VD2 TEM | VD2 TEM | 7 | (7) VD2 TEM |
| 325 | CyTOF VD2 TEM CD38+ | VD2 TEM CD38+ | 8 | (8) VD2 TEM CD38+ |
| 326 | CyTOF VD2 TEMRA | VD2 TEMRA | 9 | (9) VD2 TEMRA |
| 327 | CyTOF VD2 TEMRA CD38+ | VD2 TEMRA CD38+ | 10 | (10) VD2 TEMRA CD38+ |

**Supplementary Table 3.** Definition of 327 immunotypes used in timing and severity COVID-19 networks.
